# Encoding of social novelty by sparse GABAergic neural ensembles in the prelimbic cortex

**DOI:** 10.1126/sciadv.abo4884

**Published:** 2022-08-31

**Authors:** Zhe Zhao, Fengqingyang Zeng, Hanbin Wang, Runlong Wu, Liping Chen, Yan Wu, Shen Li, Jingyuan Shao, Yao Wang, Junjie Wu, Zhiheng Feng, Weizheng Gao, Yanhui Hu, Aimin Wang, Heping Cheng, Jue Zhang, Liangyi Chen, Haitao Wu

**Affiliations:** ^1^ Department of Neurobiology, Beijing Institute of Basic Medical Sciences, 100850 Beijing, China.; ^2^ College of Engineering, Peking University, 100871 Beijing, China.; ^3^ Academy of Advanced Interdisciplinary Study, Peking University, 100871 Beijing, China.; ^4^ State Key Laboratory of Membrane Biology, Beijing Key Laboratory of Cardiometabolic Molecular Medicine, Institute of Molecular Medicine, Peking-Tsinghua Center for Life Sciences, College of Future Technology, Peking University, 100871 Beijing, China.; ^5^ Beijing Transcend Vivoscope Biotech Co. Ltd., 100094 Beijing, China.; ^6^ State Key Laboratory of Advanced Optical Communication System and Networks, School of Electronics, Peking University, 100871 Beijing, China.; ^7^ PKU-IDG/McGovern Institute for Brain Research, 100871 Beijing, China.; ^8^ National Biomedical Imaging Center, Beijing 100871, China.; ^9^ Key Laboratory of Neuroregeneration, Coinnovation Center of Neuroregeneration, Nantong University, Nantong 226019, Jiangsu Province, China.; ^10^ Chinese Institute for Brain Research, 102206 Beijing, China.

## Abstract

Although the prelimbic (PrL) area is associated with social behaviors, the neural ensembles that regulate social preference toward novelty or familiarity remain unknown. Using miniature two-photon microscopy (mTPM) to visualize social behavior–associated neuronal activity within the PrL in freely behaving mice, we found that the Ca^2+^ transients of GABAergic neurons were more highly correlated with social behaviors than those of glutamatergic neurons. Chemogenetic suppression of social behavior–activated GABAergic neurons in the PrL disrupts social novelty behaviors. Restoring the MeCP2 level in PrL GABAergic neurons in *MECP2* transgenic (*MECP2*-TG) mice rescues the social novelty deficits. Moreover, we identified and characterized sparsely distributed NewPNs and OldPNs of GABAergic interneurons in the PrL preferentially responsible for new and old mouse exploration, respectively. Together, we propose that social novelty information may be encoded by the responses of NewPNs and OldPNs in the PrL area, possibly via synergistic actions on both sides of the seesaw.

## Teaser

Sparse GABAergic neuronal ensembles are responsible for encoding social novelty and familiarity in the prelimbic cortex.

## INTRODUCTION

Deficits in social cognition underlie various psychiatric disorders, including autism spectrum disorders (ASDs), schizophrenia, and major depression disorders (*1*–*3*). Although social awareness may differ between humans and rodents, some of the neural mechanisms underlying social behaviors are conserved between these species (*4*). Numerous studies have demonstrated that the medial prefrontal cortex (mPFC) is a critical regulator of social behavior such as motivation, recognition, dominance, and reward in many animal species (*5*–*10*). Furthermore, a subtype of mPFC neurons has been demonstrated to play an essential role in regulating social exploration. For instance, transient activation of excitatory neurons in the mPFC disrupts sociability (*11*), and dysfunction of mPFC excitatory neural ensembles is associated with abnormal social exploration (*8*). Furthermore, some mPFC excitatory neurons, including prelimbic (PrL) neurons, demonstrate enhanced activity during social behaviors in mice (*12*–*15*). Notably, mPFC neurons exhibit highly heterogeneous responses to various social contexts (*16*–*18*). Therefore, how these neurons encode relevant but distinct social information is unknown. In addition, although we know that GABAergic (gamma-aminobutyric acid-releasing) interneurons (INs) within the mPFC are associated with social behaviors (*10*, *19*–*22*), how this normal social information is processed at the single-cell level is still unknown.

Social deficits are the hallmark symptoms of autism in humans and can be mimicked in mouse models overexpressing methyl-CpG–binding protein 2 (MECP2). These mouse models all demonstrate severe autistic symptoms, including impaired social interactions (*23*, *24*). Duplications of MECP2 cause social recognition deficits, impair social novelty preference (*25*) and synaptic plasticity, and alter neural activity (*25*–*27*). Although extensive studies have been conducted on the neurodevelopmental, electrophysiological, and behavioral changes in *MECP2* transgenic *(MECP2*-TG) animals, the neural encoding mechanisms underlying social cognition impairment that are comprised in *MECP2*-TG mice remain elusive. One problem is the limited spatial resolution of currently used recording approaches, including functional magnetic resonance imaging (fMRI), electroencephalography (EEG), and single-photon microscopy (*6*, *8*, *28*–*30*). These methods cannot reveal the critical information embedded in the neuronal ensembles associated with social interactions. Recently, we developed a state-of-the-art miniature two-photon microscope (mTPM) that enables in vivo calcium imaging in freely moving animals expressing the GCaMP6s fluorescent calcium sensor (*31*, *32*), which is a powerful new tool to overcome the abovementioned challenge.

We attached an mTPM to freely behaving mice to visualize social behavior–associated neuronal activity within the PrL in a two-chamber apparatus and a three-session social behavior paradigm (*15*). In the PrL, we found more correlated activities from GABAergic INs with social behaviors than glutamatergic neurons. Chemogenetic suppression of these social behavior–activated GABAergic INs substantially disrupts the sociability and social novelty preference. Furthermore, ablation of overexpressed *MECP2* in PrL GABAergic INs by adeno-associated virus (AAV)–mediated CRISPR-Cas9 gene editing dramatically reversed social novelty exploration deficits of *MECP2*-TG mice, indicating the causal relationship. In addition, we identified two distinct sparse GABAergic neural ensembles named new and old mouse exploration–preferred INs (NewPNs and OldPNs, respectively), which responded differently in different social contexts. The activity of NewPNs and OldPNs was delayed upon encountering a new mouse compared with an old mouse, but these delayed responses were abolished in *MECP2-*TG mice. The Ca^2+^ transients of OldPNs were amplified during social with old mice compared with new mice, and this effect was also eliminated by overexpression of the *MECP2* gene. Together, our findings reveal that aberrant encoding by GABAergic neural ensembles in the PrL area is responsible for the impairment of social novelty discrimination in a mouse model of *MECP2* duplication syndrome.

## RESULTS

### Visualization of neural ensembles responsible for social interactions within the PrL area by miniature two-photon calcium imaging

**Fig. 1.**
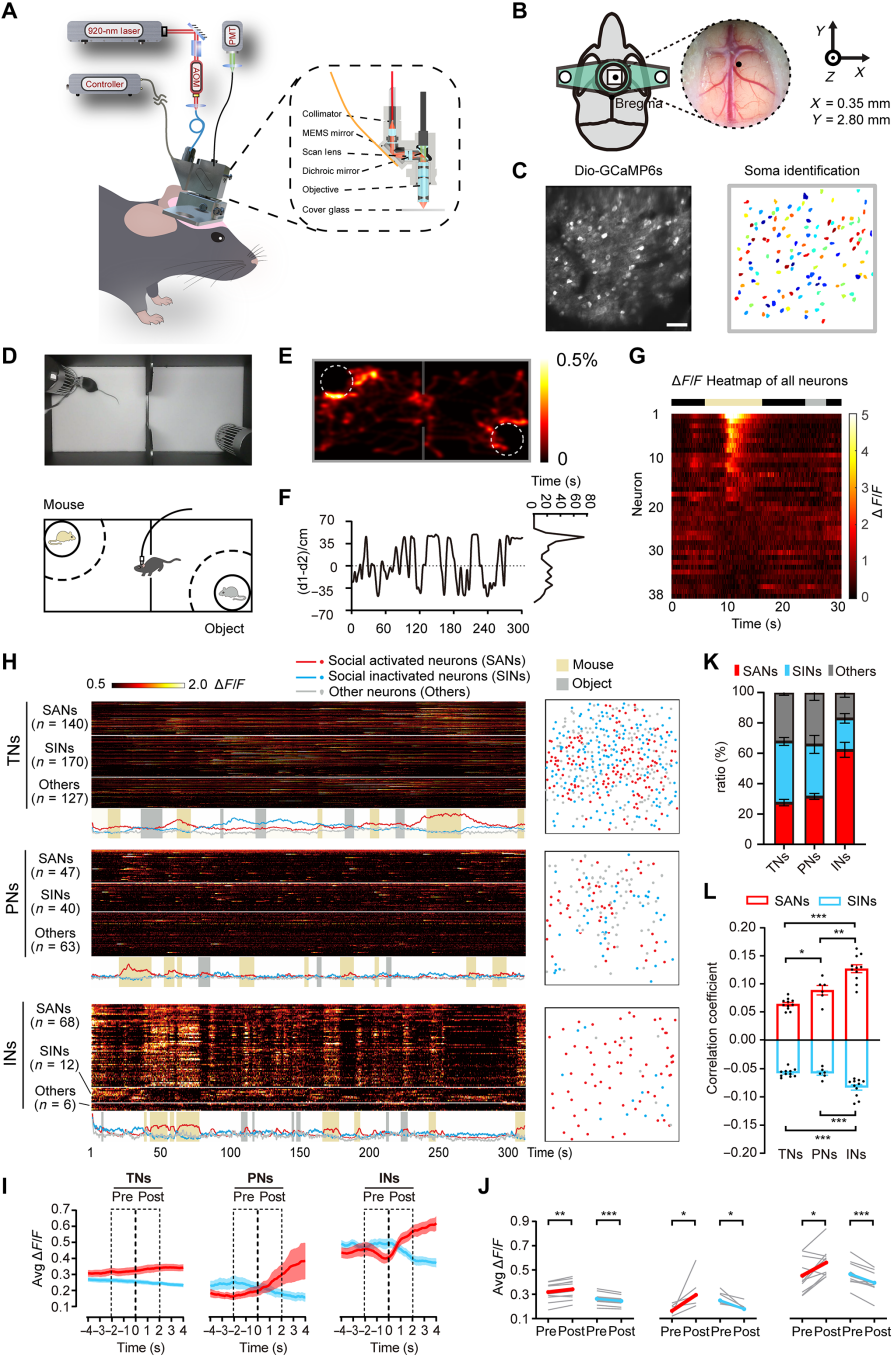
Decoding of social behaviors in distinct neural ensembles within the PrL via miniature two-photon microscopy in freely behaving mice. (**A**) Schematic diagram of the mTPM mounted on the head of a mouse. The setup of the mTPM lens is illustrated by the black dotted square. (**B**) Diagram of the head-mounted chamber. The inset shows the site of craniotomy above the PrL area, and the black dot indicates the injection site. (**C**) Representative SD projection of 3000 images showing virus-mediated expression of GCaMP6s in the PrL area. Scale bar, 60 μm. (**D** and **E**) Schematic of the two exploration chambers and a heatmap showing the movement of the mice. The color in (E) indicates the proportion of exploration time. Left circle, mouse; right circle, object. (**F**) Kymograph of the mice location in a 5-min trial. The right panel illustrates the occupancy time in the track zone. d1, distance to object; d2, distance to mouse. (**G**) Heatmap of the calcium activity of a representative mouse. The depth of the color reflects the Δ*F*/*F* value. (**H**) Heatmaps of neural calcium activity and the average responses of TNs, PNs, and INs in three mice. Neurons were clustered into SAN, SIN ensembles, and other neurons. The spatial distribution is shown on the right. (**I**) Event average Δ*F*/*F* calcium traces of SANs and SINs among TNs, PNs, and INs at social exploration onset. (**J**) Comparison of Δ*F*/*F* values before and after social exploration onset. Gray lines, individual changes; red and blue lines, average changes. (**K**) Ratios of SANs, SINs, and other neurons among TNs, PNs, and INs. (**L**) Statistical analysis of the signal-behavior correlation coefficient. *n* = 11 mice (TNs), *n* = 6 mice (PNs), and *n* = 11 mice (INs), respectively. For detailed information and statistics, see Materials and Methods and table S1.

As the brain region that critically mediates social motivation and social behaviors, the PrL area has been studied in freely behaving mice using miniature wide-field microscopes (*8*, *13*, *15*, *33*). To ensure that we were able to record the activity of individual neurons, we monitored time-dependent fluctuations in GCaMP6s signals in mice with a head-mounted mTPM (*31*, *32*, *34*) (Fig. 1, A and B), which did not significantly alter mouse behavior (fig. S1A). GCaMP6s-positive neuronal somata were segmented with the custom-developed framework based on MATLAB (Fig. 1C).

We adopted a modified two-chamber assay to study neuronal activity associated with socially conditioned place preference (*15*). We placed one mouse in one chamber and a plastic object in the other chamber (Fig. 1D), and observed both the behavior and neuronal activity of mice with a head-mounted microscope as they freely explored the field and the two opposing chambers (movie S1). Mouse trajectories and direct exploration activity were automatically detected and accompanied by manual inspection (see Materials and Methods). The results revealed that the mice explored the chamber containing the mouse more than the chamber containing the plastic object (Fig. 1E). Moreover, the mice spent more time on the side of the field with the mouse (Fig. 1F and fig. S1B), confirming that mice might prefer to socialize with a partner mouse. Next, we manually identified social interaction epochs in which the mouse actively contacted the encounter mouse in the cage by direct exploration (*8*). Representative traces of the activity of 38 INs within the PrL area of a freely behaving mouse during social and object exploration are shown in Fig. 1G and fig. S1C. A small population of socially tuned PrL neurons responded with prominent calcium transients when the mouse approached the encounter mouse within these social epochs but stayed inactive during exploring the cage containing the plastic object (Fig. 1G). Therefore, we demonstrate a paradigm for studying the social preference of mice and its associations with the activity of neural ensembles within the PrL area.

PrL neurons (TNs) include both pyramidal neurons (PNs) and GABAergic INs, which may respond to social behaviors differently. Therefore, to uncover the encoding mechanism, we infected wild-type (WT), CaMKII-Cre transgenic, and Viaat-Cre transgenic mice with AAV or Cre-dependent DIO-AAV expressing GCaMP6s to label TNs, PNs, and INs, respectively (movies S2 and S3). These mice spent more time exploring the encounter mouse than the opposite plastic object, and there were no differences among the strains (fig. S2). Then, we followed a previously published protocol to dissect activated and inactivated neurons in response to social interaction (see Materials and Methods). In short, the Ca^2+^ trace of a single neuron within one epoch of social interaction was temporarily shuffled hundreds of times to test the significance of the association of the Ca^2+^ transient with the behavior itself (fig. S3A). By calculating and comparing distinct types of neuronal calcium activity in response to various stimuli, we divided the neurons into three subtypes, i.e., socially activated neurons (SANs), socially inactivated neurons (SINs), and other neurons (Others) that were not associated with social interaction (Fig. 1H). We plotted kymographs of the activity of spatially intermixed SANs and SINs of TNs, PNs, and INs and observed a seemingly greater increase in the Ca^2+^ transients of SANs and a decrease in the Ca^2+^ transients of SINs within the IN subpopulation upon social interaction (Fig. 1H). Although SANs or SINs were found to be associated with social behaviors, the mean increase or decrease in the Ca^2+^ signals of TNs upon social interaction was small (Fig. 1, I and J), which agreed with a previous report (*8*).

According to these criteria, SANs comprised a much larger population of INs than PNs (Fig. 1K). INs exhibited elevated baseline cytosolic Ca^2+^ concentrations, while social behavior–triggered increases in Δ*F*/*F* in INs were similar to those in PNs (Fig. 1, I and J). Conversely, the average Δ*F*/*F* of SANs decreased, and that of SINs increased after the withdrawal of the social stimulus (fig. S3, B and C). We also directly tested how the Ca^2+^ responses of different neurons were associated with social behaviors (see Materials and Methods). SANs and SINs that were INs showed higher absolute correlation coefficients than PNs and TNs (Fig. 1L). The absolute correlation of Ca^2+^ signals with social behavior in INs was also the highest at the population level (fig. S3D). Moreover, SANs that were INs had the highest probability of being activated during social interaction. In contrast, SINs that were INs had the lowest probability of being activated (fig. S3E). These data show a bipolar response of Ca^2+^ activity in INs, which indicates that INs are more highly correlated with social behavior in mice.

We also constructed networks associated with social and nonsocial states to explore the functional connectivity of TNs, PNs, and INs, as previously described (*35*). Figure S4A shows representative examples of the linear connectivity of TNs, PNs, and INs in a mouse during social exploration. We quantitatively explored the neuronal network characteristics by calculating the average clustering coefficient (see Materials and Methods), including the positive and negative connectivity strengths (fig. S4B). INs showed the highest positive average clustering coefficient and the lowest negative average clustering coefficient during social exploration (fig. S4B). Together, these results indicate that INs are more strongly associated with social cues than TNs and PNs, both in the production of substantial Ca^2+^ responses at the single-cell level and in the induction of more extensive changes in network connectivity strength at the circuit level.

### Social novelty preference impairment associated with PrL neural ensembles in *MECP2*-TG mice

**Fig. 2.**
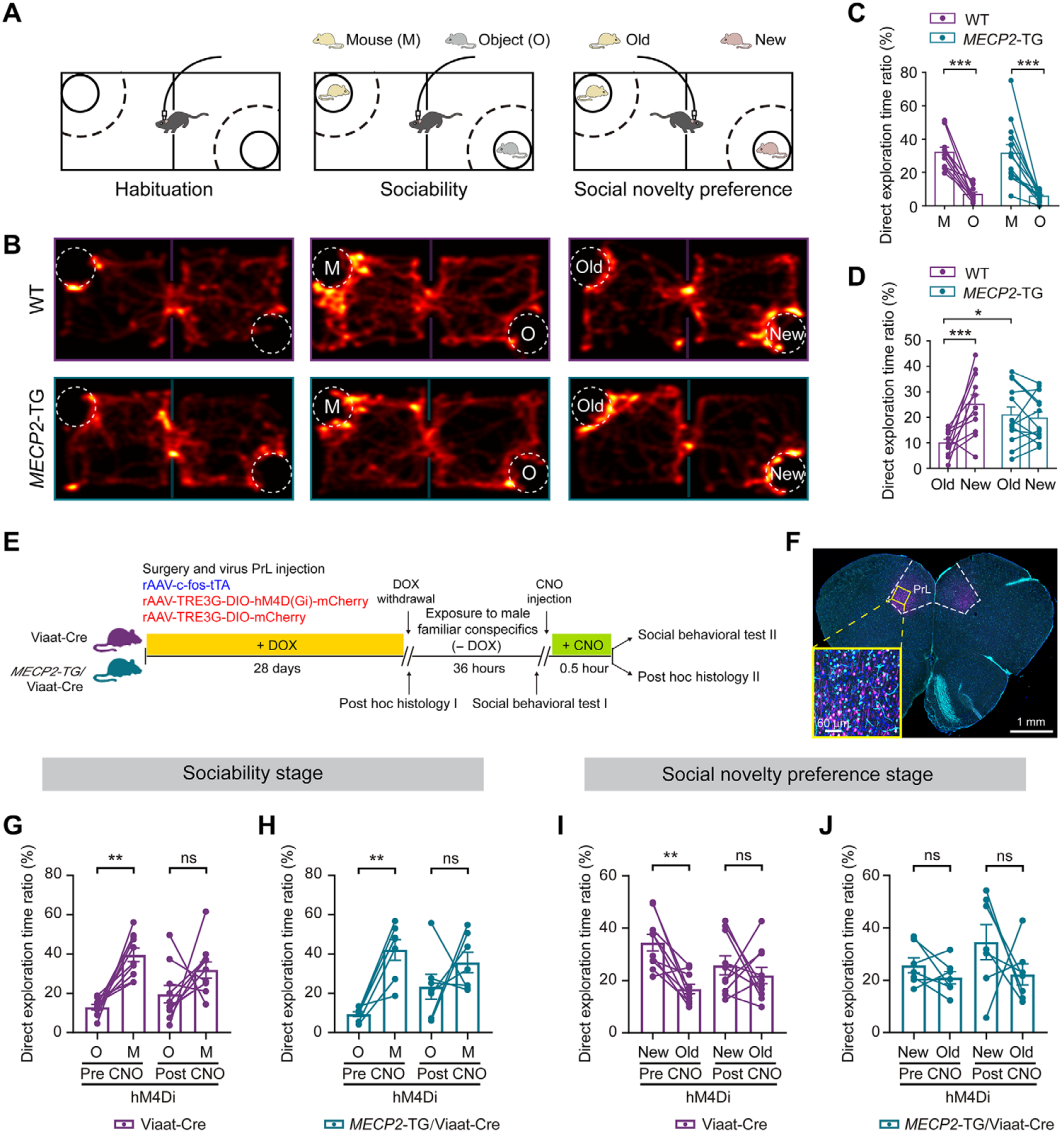
PrL neuronal ensembles are associated with the impaired social novelty preference performance in *MECP2*-TG mice. (**A** and **B**) Schematic diagram of mTPM recording in the two-chamber social test and representative heatmaps showing the movement of a WT mouse (upper) and a *MECP2-*TG mouse (lower). (**C** and **D**) Direct exploration time ratio of WT and *MECP2-*TG mice in the sociability and social novelty preference stages. (**E**) Experimental paradigm used to test the causal role of GABAergic social behavior representations in WT and *MECP2-*TG mice. Doxycycline (Dox) was administered via the drinking water at 1 g/liter for 4 weeks. (**F**) Representative image showing activated GABAergic INs in the PrL. Magenta, mCherry-positive neurons involved in exploring home cage conspecifics; cyan, Viaat-Cre–positive GABAergic INs. (**G** and **H**) Direct exploration time ratio with the stranger mouse (M) and object (O) for WT and *MECP2*-TG mice before and after CNO administration. (**I** and **J**) Direct exploration time ratio with the new mouse (new) and old mouse (old) for WT and *MECP2*-TG mice before and after CNO administration. The data are shown as the mean ± SEM. For detailed information and statistics, see Materials and Methods and table S1.

Human *MECP2*-TG mice mimicking the broad spectrum of phenotypes observed in ASD patients, such as deficiency of social interactions, were generated (*23*). Normal mice can discriminate between new and familiar mice (*36*), while *MECP2*-TG autism model mice cannot (*23*, *25*, *37*, *38*). Therefore, following the sociability test, we performed a social novelty preference assay in which the plastic object (O) was replaced with a new mouse (M) (Fig. 2A). Representative heatmaps of the trajectories of the mice are shown in Fig. 2B. During the sociability test, both the WT mice and the *MECP2*-TG mice spent a significantly longer time exploring the mouse (M) than the plastic object (O) (Fig. 2C and fig. S5). However, while WT mice spent more extended periods exploring the new mouse in the social novelty test, the *MECP2*-TG mice showed no preference between the old mouse and the new one (Fig. 2D and fig. S5). These studies further confirm that social novelty preference is impaired in *MECP2*-TG mice, which agrees with a previous study (*25*).

Next, to explore the causal relationship between social exploration behaviors and GABAergic neuronal activity in the PrL, we used a chemogenetic suppression approach under the control of the Tet-Off system (Fig. 2E). Briefly, we simultaneously coinjected activity-dependent rAAV-c-fos-tTA-WPRE-pA and rAAV-TRE3G-DIO-hM4D(Gi)-mCherry-WPRE-pA into the PrL region of the mouse brain (Fig. 2E and fig. S6A). Because the TRE3G was occluded by the exogenous doxycycline (Dox) (fig. S6A), this allowed us to specifically restrict the expression of hM4Di to social exploration–activated GABAergic neural ensembles in Viaat-Cre mice (Fig. 2F and fig. S6B). Subsequently, social preference could be assessed in a two-chamber social behavior assay once GABAergic SANs in the PrL area were selectively suppressed (Fig. 2, G to J, and fig. S6, C and D). When SAN neural ensembles were suppressed in mice infected with hM4Di-expressing virus after clozapine *N*-oxide (CNO) administration, social preference was abolished in both Viaat-Cre and *MECP2*-TG/Viaat-Cre mice (Fig. 2, G and H). In contrast, behavior performances were not affected in rAAV-TRE3G-DIO-mCherry-WPRE-pA control virus–injected Viaat-Cre and *MECP2*-TG/Viaat-Cre mice, respectively (fig. S6, C and D). While similar treatments disrupted social novelty preference in Viaat-Cre control mice (Fig. 2I), *MECP2* overexpression compromised the preference for socially novel mice independent of chemogenetic suppression (Fig. 2J). Overall, these studies demonstrate that some GABAergic SANs in the PrL participate in sociability and social novelty preference, whereas overexpressing *MECP2* may impair social novelty by disrupting the activity of functional ensembles of INs in the PrL region.

### Restoration of social novelty behavior in *MECP2*-TG mice through CRISPR-Cas9–based gene editing of PrL GABAergic neurons

**Fig. 3.**
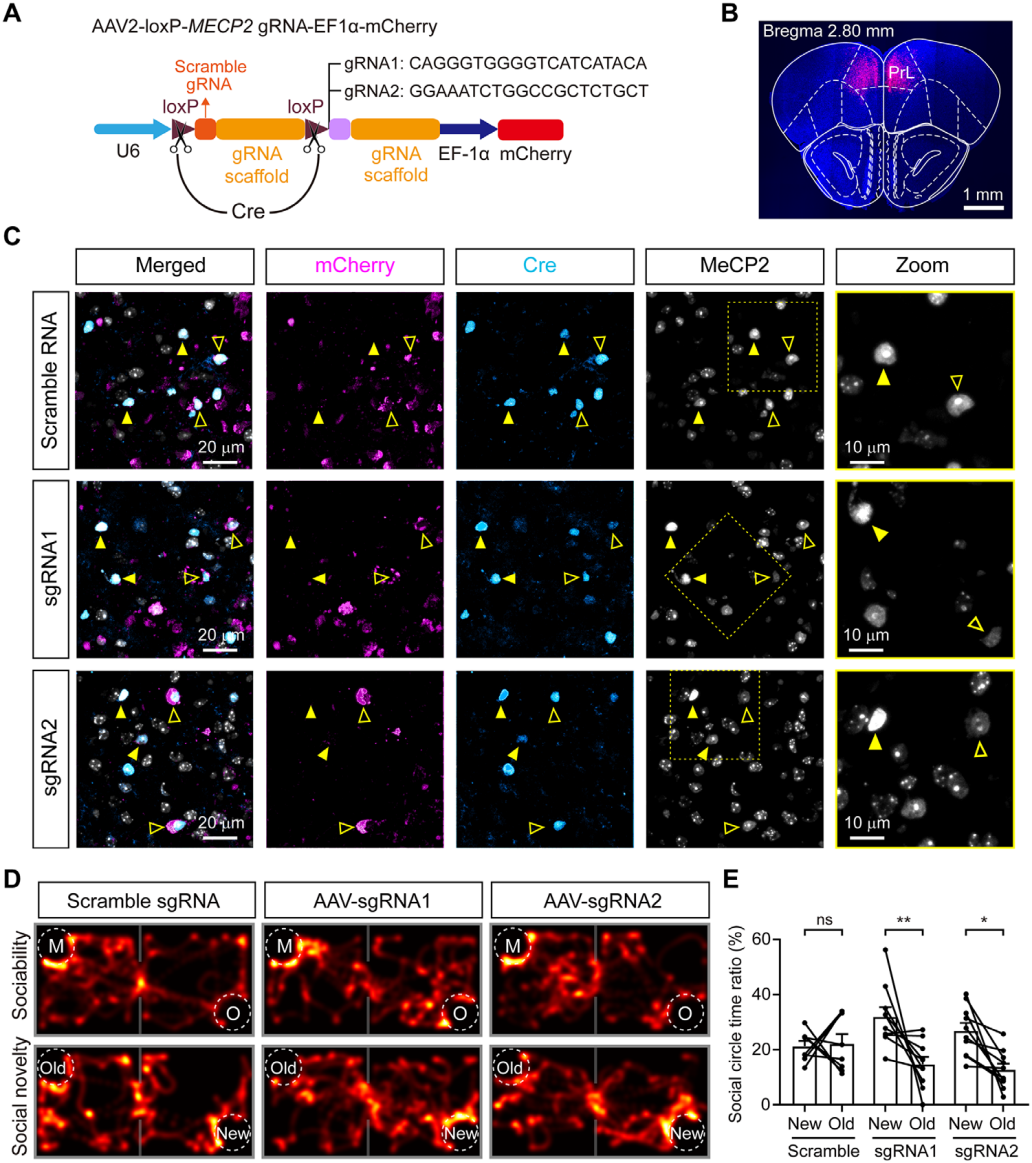
Restoring MeCP2 levels within the PrL INs via CRISPR/Cas9 reverses the social novelty deficit in *MECP2*-TG mice. (**A**) Schematic diagram of the AAV expression vectors. sgRNAs targeting the human *MECP2* gene were used. (**B**) Representative image indicating the location of viral expression after injection. Red: mCherry fluorescence in the PrL. (**C**) Representative images of brain slices from *MECP2*-TG/Viaat-Cre/Rosa26-Cas9 triple transgenic mice immunostained with MeCP2 antibody. Viral infected and noninfected INs were identified by crossing anti-Cre immunofluorescence and mCherry expression (indicated by hollowpoint arrowheads and arrowheads, respectively). AAV-sgRNA1– and AAV-sgRNA2–infected INs show significantly reduced expression of MeCP2 compared to scramble RNA–infected INs. (**D**) Representative heatmaps showing the movement of *MECP2*-TG mice injected with scramble sgRNA (left), *MECP2*-TG mice injected with AAV-sgRNA1 (middle), and *MECP2*-TG mice injected with AAV-sgRNA2 (right) in the sociability and social novelty preference stages. (**E**) Normalizing MeCP2 levels in the mPFC reversed impaired social novelty preference recognition behavior in the two-chamber test. The data are shown as the mean ± SEM. For detailed information and statistics, see Materials and Methods and table S1.

Previous works have demonstrated that abnormal social behaviors can be reversed in adult *MECP2*-TG mice by normalization of MeCP2 levels throughout the whole brain, in the mPFC, and in the hippocampal CA1 region (*25*, *27*, *39*). To specifically delete the exogenous human *MECP2* transgene in PrL GABAergic INs, we designed a Cre-dependent AAV-based construct by inserting loxP sites on either side of a U6 promoter–driven scramble guide RNA (gRNA) followed by a single-guide RNA (sgRNA) specifically targeting exon 3 of the human *MECP2* gene and EF1a promoter–driven mCherry (termed AAV2-loxP-MECP2-gRNA-EF1a-mCherry) (Fig. 3A). CRISPR-Cas9–mediated human *MECP2* gene knockout efficiency was confirmed in transfected human embryonic kidney (HEK) 293T cells (fig. S7, A and B), while the expression of mouse *Mecp2* gene was not affected (fig. S7, C and D). We crossed *MECP2*-TG mice with Viaat-Cre mice and then crossed the offspring with Rosa26-Cas9 knock-in mice harboring a constitutively expressed Cas9 allele (fig. S8A) (*40*). Subsequently, we bilaterally delivered AAV2-loxP-MECP2-gRNA-EF1a-mCherry carrying sgRNA1 or sgRNA2 (termed AAV-sgRNA1 and AAV-sgRNA2, respectively) and scramble RNA virus into the PrL region of adult *MECP2*-TG mice (Fig. 3B). The suppressive efficiency of human MECP2 expression in mCherry^+^ GABAergic INs within the PrL subregion was validated by immunostaining with anti-MeCP2 antibody 4 weeks after AAV-sgRNA virus infection (Fig. 3C). Strikingly, both AAV-sgRNA1– and AAV-sgRNA2–injected *MECP2*-TG mice spent more time interacting with the new mouse than the familiar mouse (Fig. 3, D and E). In contrast, scramble RNA–injected *MECP2*-TG mice still failed to discriminate between the new and old social objects (Fig. 3, D and E), although the performance was not affected in both habituation and sociability stages as shown previously (Figs. 2, A to C, and 3D and fig. S8, B and C). Therefore, while abnormal social novelty behavior in *MECP2*-TG mice may be mainly due to disruption of PrL GABAergic INs, normalization of *MECP2* levels in PrL GABAergic INs can dramatically rescue social novelty defects in *MECP2*-TG mice.

### Identification and characterization of social cue–preferred neural ensembles within the PrL area in WT and *MECP2*-TG mice

**Fig. 4.**
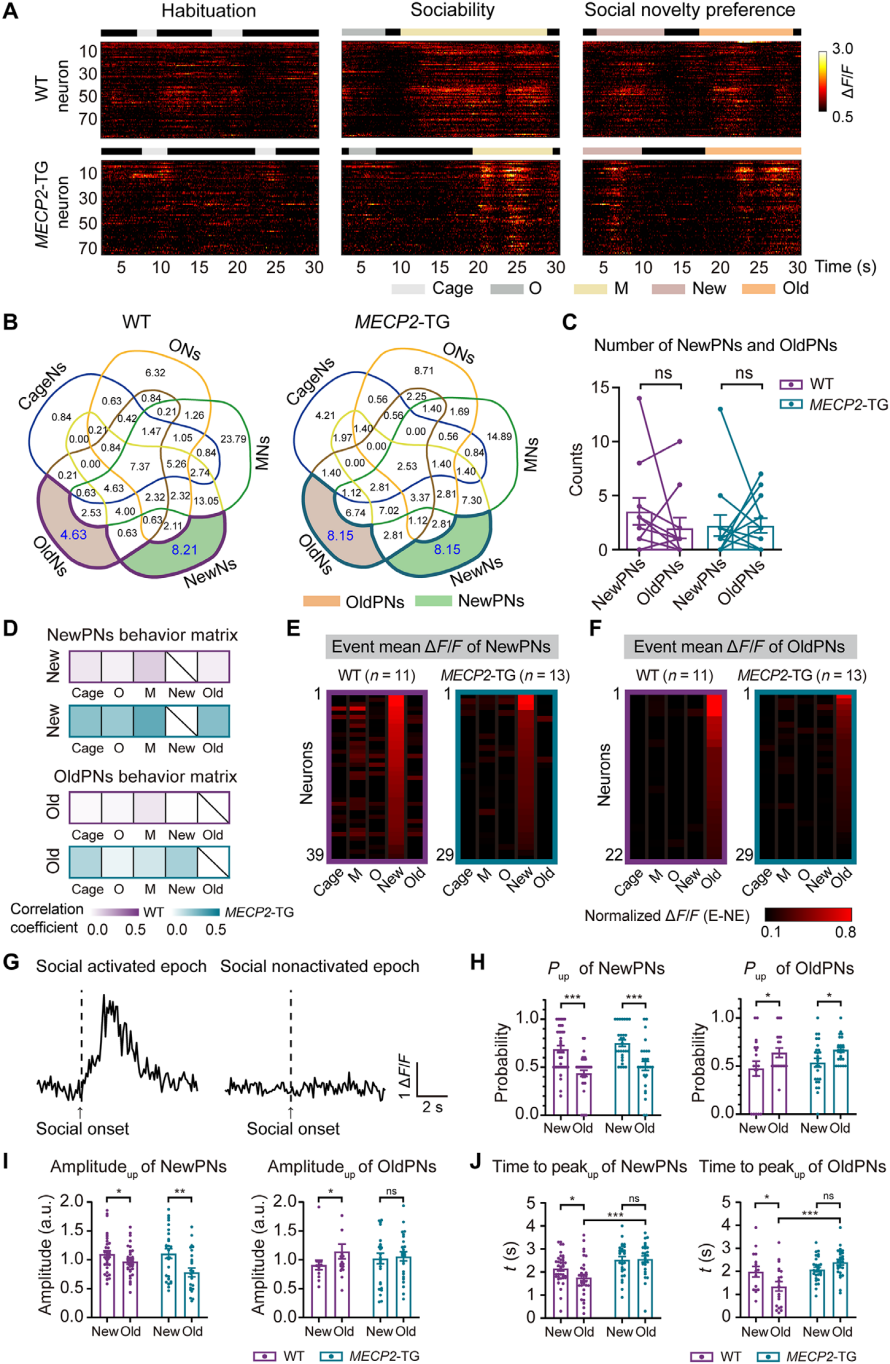
Identification and characterization of GABAergic NewPNs and OldPNs within the PrL in WT and *MECP2*-TG mice. (**A**) Representative heatmaps of INs in WT and *MECP2*-TG mice at different stages. (**B**) Venn diagram illustrating the relationship between EANs, NewPNs, and OldPNs. Each colored leaf represented one kind of EAN. The number inside lattice: the ratio (units: %) of this type among all EANs. Left: WT mice, *n* = 475 neurons. Right: *MECP2*-TG mice, *n* = 356 neurons. (**C**) Number of NewPNs and OldPNs. Purple, WT mice (*n* = 11); green, *MECP2*-TG mice (*n* = 13). (**D**) Average behavioral correlation matrix of the calcium activity of NewPNs and OldPNs during different exploration behaviors. Purple, WT mice (*n* = 11); green, *MECP2*-TG mice (*n* = 13). (**E** and **F**) Heatmaps of the event mean Δ*F*/*F* values of NewPNs and OldPNs in five different exploration states. Intensity was normalized by the maximum value across all exploration states. (E) NewPNs in WT mice (*n* = 39) and *MECP2*-TG mice (*n* = 29), respectively; (F) OldPNs in WT mice (*n* = 22) and *MECP2*-TG mice (*n* = 29), respectively. (**G**) Typical calcium traces of socially activated epoch and socially nonactivated epoch. (**H**) Activation probability of NewPNs and OldPNs in all new and old mouse exploration epochs. Any social cue–preferred IN that did not show an activated epoch under its corresponding social cue was eliminated. (**I**) Extent of the increase of NewPNs and OldPNs in socially activated epochs. All values were normalized by the mean value under social cue–activated epochs. a.u., arbitrary units. (**J**) Rise time of NewPNs and OldPNs in socially activated epochs. The data are shown as the mean ± SEM. For detailed information and statistics, see Materials and Methods and table S1.

To further examine the neural coding mechanisms underlying social novelty preference, we focused on the calcium dynamics of INs in the PrL cortex in WT and *MECP2*-TG mice exposed to different exploration cues (Fig. 4A). By calculating the differences between the mean amplitude during exploration and nonexploration (NE), we established a criterion for identifying neural ensembles associated with specific cues (see Materials and Methods). Neurons with a mean relative amplitude β times higher than the noise level of the NE state were considered event-associated INs (EANs), including cage exploration–associated INs (CageNs), object exploration–associated INs (ONs), mouse exploration–associated INs (MNs), and new or old mouse exploration–associated INs (NewNs or OldNs, respectively). At β = 0.5, activated ensembles comprised ~50% of INs in both WT and *MECP2*-TG mice (fig. S9). While the ratio of MNs was higher than that of ONs in both WT and *MECP2*-TG mice, the percentage of NewNs was much higher than that of OldNs in WT, but not *MECP2*-TG, mice in the social novelty test (fig. S10A), which is consistent with the aforementioned social behavior results (Fig. 2D).

Paradoxically, the mean event-evoked Ca^2+^ amplitudes and dynamics of specific EANs were similar between WT and *MECP2*-TG mice (fig. S10, B and C). We wondered whether this was due to the nonpreferential activation of EANs by different behaviors. By calculating the average behavioral correlation matrix (see Materials and Methods), we found that NewN, OldN, CageN, MN, and ON neural ensembles responded to multiple behaviors without preference (fig. S11, A and B). In addition, the ratios of OldNs or NewNs were not significantly different between WT mice and *MECP2*-TG mice, which raised the possibility that only a subset of OldNs and NewNs are affected by *MECP2* overexpression (fig. S10A). Given that every mouse underwent three different exploration paradigms, we wondered whether we could dissect neurons that preferentially responded to one type of cue. In 11 WT mice, 475 of 817 neurons were EANs, and 356 of 788 neurons were EANs in 13 *MECP2*-TG mice (fig. S12). While most neurons were classified into more than one EAN subtype, some were categorized as NewNs or OldNs only (fig. S12). We used a modified Venn diagram to visualize the intricate intersection relationships (http://bioinformatics.psb.ugent.be/webtools/Venn/) (Fig. 4B). Neurons that preferentially responded to old or new mice were named new or old mouse exploration–preferred INs (NewPNs and OldPNs, respectively). We detected 39 NewPNs (~8.21% of all responsive neurons) and 22 OldPNs (~4.63%) in 11 WT mice and 29 NewPNs (~8.15%) and 29 OldPNs (~8.15%) in 13 *MECP2*-TG mice (Fig. 4, B and C).

As shown in the behavioral correlation matrix, compared to NewNs and OldNs, NewPNs and OldPNs demonstrated much weaker responses to other nonpreferential social cues in both WT and *MECP2-*TG mice (Fig. 4D, fig. S11B, and movies S4 to S7). Therefore, during all new and old mouse exploration events, NewPNs responded prominently to the new mouse, exhibiting a robust increase in Ca^2+^ signals, but rarely responded to the old mouse in WT and *MECP2*-TG mice (Fig. 4E and movies S4 and S6). In contrast, we observed that the preference of OldPNs to respond to the old mouse was compromised in *MECP2*-TG mice, as the new mouse evoked a relatively more robust activation than did WT mice (Fig. 4F and movies S5 and S7).

To pinpoint the effects of *MECP2* overexpression on the activity of specific neural ensembles, we examined the Ca^2+^ dynamics of NewPNs and OldPNs in socially activated epochs upon exposure to either the new mouse or old mouse (see Materials and Methods). While social cues often evoked robust Ca^2+^ transients in these neurons, they were occasionally nonresponsive (Fig. 4G). Specifically, in contrast to a greater probability of activation under specific preferred social cues, NewPNs and OldPNs were randomly activated with a likelihood of ~50% under the nonpreferred social cues (Fig. 4H). These properties were maintained in both WT and *MECP2*-TG mice. In contrast, the preferred activation of the OldNs (exclude OldPNs) by different social contexts was insignificant in WT mice and unaffected by *MECP2* overexpression (fig. S13A). However, in these socially activated epochs, the old mouse evoked a significant increase in Ca^2+^ transient amplitudes compared with the new mouse in OldPNs in WT mice but not in *MECP2*-TG mice (Fig. 4I). Last, we found that the amplitude of Ca^2+^ transients of both NewPNs and OldPNs evoked by exposure to the new mouse peaked later than those elicited by the old mouse in WT mice but that this effect disappeared in *MECP2*-TG mice (Fig. 4J). However, the aforementioned differences in neuronal preference to different social contexts of both WT and *MECP2*-TG mice were indistinguishable in NewNs (except NewPNs) and OldNs (except OldPNs) (fig. S13). These data reinforce that distinctive and sparse neural ensembles are responsible for preferentially encoding novel and old social information.

Next, we detected and characterized the social cue–preferred GABAergic NewPNs and OldPNs in AAV-sgRNA–mediated *MECP2*-TG rescue mice (*MECP2*-TG res) (Fig. 3, A to C, and fig. S14, A and B). Analysis of calcium dynamics in socially activated epochs revealed that delayed responses of NewPNs upon exposure to old mice were accelerated and rectified in *MECP2*-TG res mice (fig. S14, C and E). The dysfunction of OldPNs was also partially recovered, although reaction amplitudes between new and old social activated epochs were still indistinguishable (fig. S14D); the time spent under old mice exposure was significantly rescued to normal compared to controls (fig. S14F). Overall, the functional balance of social cue–preferred INs was markedly reconstructed in *MECP2*-TG res mice with a recovered preference for new partners after restoring MeCP2 expression in PrL GABAergic INs.

Together, these results indicate dysfunction of a small population of social cue–preferred neural ensembles that respond to both new and old mice with delayed Ca^2+^ transients in *MECP2*-TG mice. Moreover, novelty cue–preferred INs, which are present in WT mice, respond nonpreferentially to new and old mice in *MECP2*-TG mice. Ultimately, this disrupted balance may casually lead to an inability to discriminate between new and old social objects in *MECP2*-TG mice (Figs. 2, B and D, and 4 and fig. S14).

## DISCUSSION

Most animals, including humans, live within social groups in which they interact with many other group members (*5*). The neuronal coding mechanisms that mediate social identity recognition and group behavior are esoteric and cutting-edge scientific questions (*41*). Social novelty is a concept that has been proposed along with the development of the three-step behavior paradigm (*36*), which has frequently been used to assess social dysfunction in genetic mouse models of ASD (*4*, *42*). Using a mini-epifluorescence microscope, Liang *et al.* (*8*) identified an excitatory neuronal ensemble encoding social exploration and novelty in the mPFC region of mice and showed that ~30% of SANs were associated with social novelty recognition. Our experiment confirmed this conclusion using a head-mounted mTPM (Fig. 1K). Moreover, we found that a higher proportion of INs than PNs was SANs in the PrL area, as they demonstrated higher calcium activity in response to social cues (Fig. 1K and fig. S3D). However, the level of involvement of PNs in the PrL area in social interaction that we observed was much lower than previously reported (fig. S3E). This may be due to contaminations from out-of-focus emissions in epifluorescence images, which makes the identification of individual neurons difficult. The advantage of two-photon excitation was demonstrated by our ability to capture a much higher proportion of socially associated neurons that were GABAergic INs despite their lower overall abundance (Fig. 1K). Correspondingly, these individual INs demonstrated amplified and correlated socially elicited Ca^2+^ transients (Fig. 1, I and J). In combination with the outcomes of activity-dependent chemogenetic manipulation (Fig. 2), these data confirm PrL GABAergic INs in mediating social preference (*4*, *19*, *21*, *43*).

**Fig. 5.**
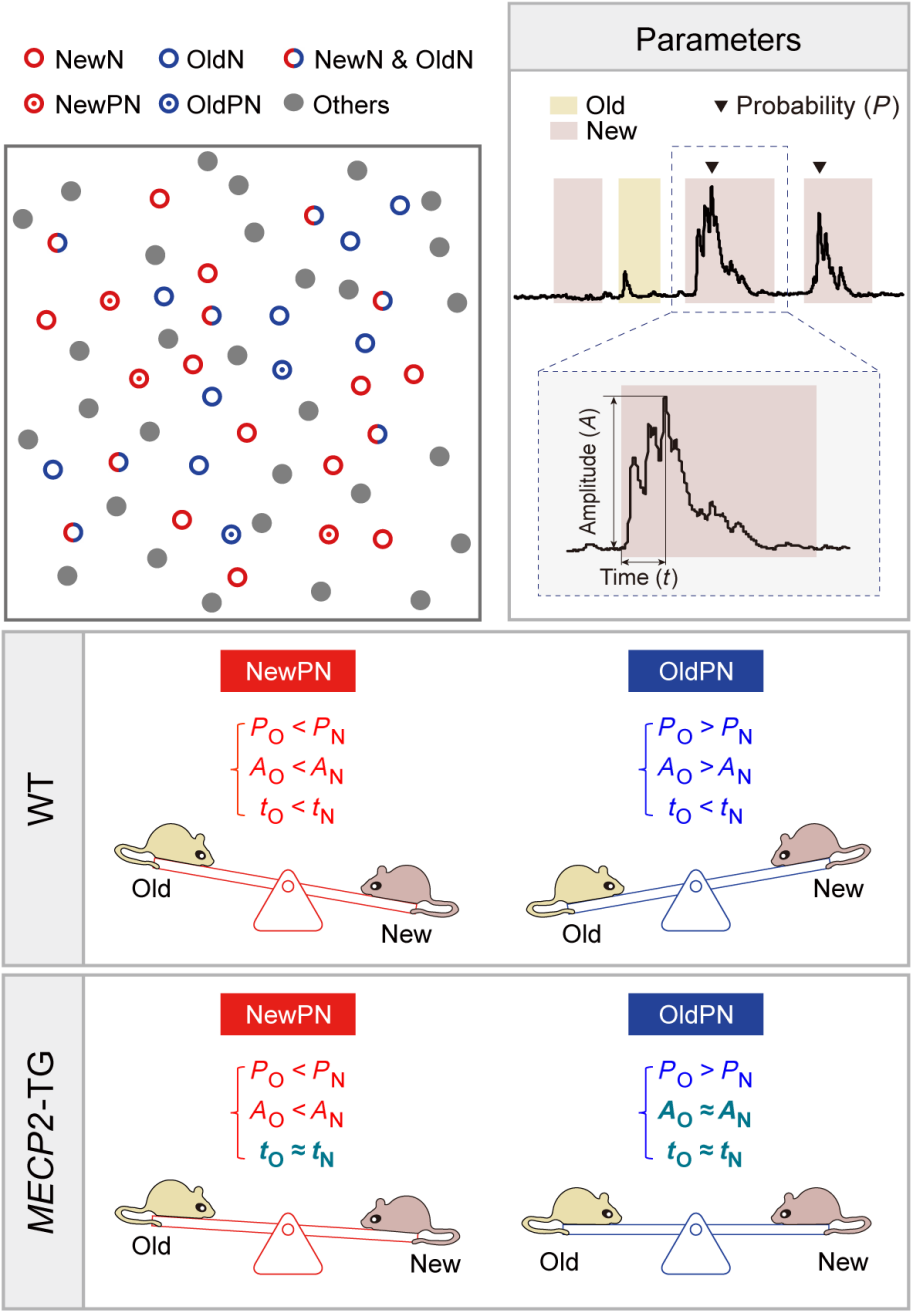
Working model for sparse GABAergic neural ensembles encoding social novelty. In the prelimbic (PrL) cortex, NewPNs and OldPNs may confer social novelty and familiarity on two sides of the seesaw, while duplication of MECP2 profoundly disrupts the balance via synergistic actions.

In previous studies, neurons evoked by social cues were often defined by animal behaviors in the sociability stage only (*15*, *44*, *45*). However, we showed here that these neurons also respond to other cues in the habituation and social novelty preference phases (Fig. 4A). GABAergic INs send multiple inputs to excitatory neurons in the PrL (*10*, *16*, *46*), and the PrL also receives various long-range inputs from other cortical and subcortical areas, which may involve signal modulation and integration (*47*, *48*). It is conceivable that NewNs and OldNs among PrL GABAergic INs may modulate social behaviors by directly regulating PN activity or indirectly regulating their long-range downstream subcortical projections (*15*, *44*, *49*, *50*). To enable behavioral coherence with the complexity of neuronal networks, we incorporated the neuronal activity and behaviors of mice into three stages and developed a new classification of neurons that show a preference for social exploration (NewPNs and OldPNs). Compared to NewNs and OldNs, these NewPNs and OldPNs were sparse (Fig. 4C) and encoded novelty/familiarity information much more precisely (Fig. 4, E and F). Despite the selective deficiency in social novelty exploration exhibited in *MECP2*-TG mice (Fig. 4, H and J), the neuronal activity of their NewNs and OldNs was not significantly different from those of control mice (fig. S13). In contrast, both NewPNs and OldPNs showed faster increases in Ca^2+^ transient amplitudes upon encountering old social cues than upon exposure to new cues, which were compromised in *MECP2-*TG mice (Fig. 4J) and could be rescued after restoring MeCP2 level (fig. S14, C to E). Compared to new mice, old mice evoked more significant increases in Ca^2+^ transient amplitudes in OldPNs in WT mice, and these increases in amplitudes were diminished in *MECP2*-TG mice (Fig. 4I). In this sense, NewPNs and OldPNs may confer social novelty and familiarity on two sides of the seesaw, while MECP2 duplication profoundly disrupts the balance via synergistic actions (Fig. 5).

One interesting deduction is that novelty and familiarity information may not be encoded in isolation from each other. Both NewPNs and OldPNs were likely to respond to new and old social exploration events (Fig. 4H). The Ca^2+^ transient amplitudes of both NewPNs and OldPNs peaked later upon interaction with the new mouse than the old one (Fig. 4J). Thus, the amount of information required to process social familiarity should be lower than that required to process novelty. Given its comprehensive connectivity with temporal regions such as the hippocampus and amygdala, which are known to respond to the characteristic features of other regions (*15*, *45*, *50*), the PrL area may particularly be suited for holding representations of specific properties and play a core role in orchestrating social novelty exploration. Strikingly, the social novelty deficits observed in *MECP2-*TG mice seemed to be reversed by CRISPR-Cas9–mediated inhibition of exogenous MECP2 gene expression specifically in PrL GABAergic INs in adult mice with MECP2 duplication (Fig. 3). AAV-sgRNA1– and AAV-sgRNA2–treated *MECP2*-TG mice showed normal sociability behaviors (Fig. 3, D and E, and fig. S8), illustrating the feasibility of AAV-mediated CRISPR-Cas9 gene editing in vivo. In conclusion, our findings provide not only new insights into the coding mechanisms underlying social novelty preference in both normal and *MECP2-*TG mice but also possible therapeutic avenues for autism in vivo.

## MATERIALS AND METHODS

### Mouse strains and genotyping

All animal experiments were conducted and approved by the Institutional Animal Care and Use Committee at Beijing Institute of Basic Medical Sciences based on the *Guide for the Care and Use of Laboratory Animals* (Eighth Edition, NHR). The animals used for the experiments are 3- to 5-month-old male mice with various genotypes in the C57BL/6J background. The mice were group-housed (four to five per cage) on a 12-hour light/12-hour dark cycle (lights on from 6:30 a.m. to 6:30 p.m.) in a temperature- and humidity-controlled housing facility. Food and water were available ad libitum. Viaat-Cre transgenic mice (The Jackson Laboratory, stock no. 017535) were given by C. Zhang at Capital Medical University (*51*). Human MECP2-overexpressing mice (*MECP2*-TG, the Jackson Laboratory, stock no. 008679) were a gift from Z. Qiu at the Center for Excellence in Brain Science and Intelligence Technology, CAS (*23*). Cas9 knock-in (Rosa 26-Cas9) mice (The Jackson Laboratory, stock no. 024858) were provided by Y. Jia at Tsinghua University (*40*). Mice expressing Viaat-Cre, *MECP2*, and Rosa26-Cas9 were intercrossed to generate *MECP2*-TG*/*Viaat-Cre double and *MECP2*-TG/Viaat-Cre*/* Rosa26-Cas9 triple transgenic mice, respectively. Polymerase chain reaction (PCR)–based genotyping was performed on approximately postnatal day (P21) according to the guidelines provided by the Jackson Laboratory. Males were used in all experiments, and surgery was performed between P98 and P112.

### Generation of AAV2-loxP-MECP2 gRNA-EF1a-mCherry virus

The AAV2-loxP-EF1a-mCherry vector was a gift from Y. Jia at Tsinghua University. The pCAG-mCherry-Cre plasmid was a gift from Sbo-bio. The LentiV-Cas9-puro construct was a gift from X. Duan at Beijing Institute of Pharmacology and Toxicology. Two pairs of gRNAs targeting human *MECP2*, but not mouse *Mecp2*, were selected using the CRISPick design tool (https://portals.broadinstitute.org/gppx/crispick/public). The sequences of gRNA1 and gRNA2 were CAGGGTGGGGTCATCATACA and GGAAATCTGGCCGCTCTGCT, respectively. Oligos were synthesized and inserted into the AAV2-loxP-EF1a-mCherry vector. Correct construction of the two plasmids was verified by sequencing.

### Cell lines and validation of AAV-sgRNA knockout efficiency

HEK293T cells [American Type Culture Collection (ATCC) CRL-11268] were cultured in Dulbecco’s modified Eagle’s medium (DMEM) (Gibco, C11995500BT) supplemented with 10% fetal bovine serum (FBS; Gibco, 10091148) in a 37°C incubator with a humidified, 5% CO_2_ atmosphere. C3H/10T1/2 cells (clone 8; NIH-3T3) (ATCC CCL-226) were cultured in Basal Medium Eagle (BME) (Gibco, 41010109) supplemented with 10% FBS (Gibco, 10091148), 2 mM l-glutamine (ATCC 30-2214) in a 37°C incubator with a humidified, 5% CO_2_ atmosphere. NIH-3T3 cells were purchased from ATCC.

To confirm gRNA-mediated MECP2 knockout, NIH-3T3 and HEK293T cells were transfected with a combination of the LentiV-Cas9-puro, pCAG-mCherry-Cre (Cre), and AAV2-loxP-MECP2 gRNA1-EF1a-mCherry (gRNA1) or AAV2-loxP-MECP2 gRNA2-EF1a-mCherry (gRNA2) plasmids (for details, see table S2) for 48 hours using Lipofectamine 2000 (Invitrogen, 11668019) according to the manufacturer’s protocol. All groups of cells were transfected with the LentiV-Cas9-puro plasmid. The transfected cells were then harvested, and protein samples were prepared and subjected to Western blotting.

### Stereotaxic virus injection

Mice were anesthetized by intraperitoneal injections of pentobarbital (1 mg/kg body weight) immediately before surgery, and anesthesia was supplemented as necessary based on the hind leg reflex. The eyes of the mice were protected with ophthalmic lubricant (Puralube Vet Ointment, Chenxin Pharmaceuticals). All surgery tools and materials and the coats of the investigators were sterilized by autoclaving. The surgical area and materials that could not undergo autoclaving were sterilized by ultraviolet radiation for more than 20 min. Aseptic procedures were applied during surgery. Body temperature was maintained using a heating pad. The mice were placed in a stereotaxic apparatus (Stoelting Instruments or RWD Life Science), and the skull was leveled along both the antero-posterior and medio-lateral axes. A ~1-mm-diameter craniotomy was made unilaterally above the target area. An injection pipette was pulled from a glass tube (Sutter glass with filament) to a sharp taper (P-1000 Flaming/Brown Micropipette Puller, Sutter Instruments; tip diameter: 40 μm). A syringe pump (Stoelting Instruments) was used to inject the virus at a speed of 50 nl/min. The virus was stereotactically injected in the right PrL cortex of each mouse [+2.80 anteroposterior (AP), ±0.33 mediolateral (ML), +0.30 dorsoventral (DV)]. To target most of the PrL area, 450 nl of the virus was unilaterally delivered into a single injection site in the PrL area. After each injection, the pipette was left in place for at least 15 min before being slowly withdrawn to prevent backflow of the virus. The mice were allowed to recover on a heating pad and administered ketoprofen (Fort Dodge Animal Health) as an analgesic (5 mg/kg). An antibiotic (ampicillin sodium, 20 mg/ml, 160 mg/kg body weight) was intraperitoneally injected each day for the following three consecutive days before recordings began.

### Surgical preparation

For in vivo imaging, we unilaterally injected rAAV-hSyn-GCaMP6s-WPRE-pA (for the WT group) or rAAV-EF1a-DIO-GCaMP6s-WPRE-pA (for the CaMKII-Cre or Viaat-Cre mice) (for details, see table S2) into the right PrL area. Three to 4 weeks after virus injection, the mice were anesthetized, the head was shaved, and a circular incision was made to expose the skull over the target area. The periosteum over the exposed surface was removed using a surgical knife blade, and a homemade metal chamber was adhered to the skull with cyanoacrylate and reinforced using a mixture of cyanoacrylate adhesive and dental acrylic cement. There was a hole (7.00 mm in diameter) in the center of the chamber through which imaging was performed. The virus injection site was aligned to the center of the adapter hole. The animals were allowed to recover for 3 to 5 days after the metal chamber has adhered to the skull. Ketoprofen (5 mg/kg) and ampicillin sodium (160 mg/kg) were administered. Then, the mice were placed in a head restraint apparatus, and an approximately 4 mm by 4 mm square craniotomy centered above the PrL cortex was carefully made (tip diameter, 0.5 mm) at the appropriate stereotactic coordinates. The dura was removed, and a small piece of glass coverslip (0.13 to 0.17 mm thick, approximately 4 mm by 4 mm; Sutter Instruments) was placed on the craniotomy window. The body weight of the animals was measured everyday to monitor the recovery process. Mice were excluded if their body weight fluctuated by more than 10%.

### Miniature two-photon in vivo calcium imaging

One week after recovery from craniotomy, a benchtop two-photon microscope (FVMPE-RS, Olympus) was used to confirm the region of viral infection. To familiarize the mice with the imaging and behavior device, each mouse was allowed to fixate to the skull for 30 min per day for 3 days before the experiments to reduce anxiety during the imaging process. On the day of the behavior experiment, the mice were allowed to adapt to the training environment for 5 min before the behavioral test began, while the fiber optic was suspended with a helium balloon. Fluorescence transients in cortical neurons were monitored using a commercially available mTPM (FHIRM-TPM V2.0, Beijing Transcend Vivoscope Biotech Co. Ltd., China) equipped with a femtosecond fiber laser (Transcend Vivoscope, China) at 930 nm through a 3× water immersion objective (0.5 NA, 1-mm working distance; Transcend Vivoscope, China). The emission filter was 500 to 550 nm for GCaMP6s. Scanning and image acquisition were performed using imaging software (GINKGO-MTPM, Transcend Vivoscope, China). Frames of 512 × 512 pixels were acquired at a rate of 9.76 Hz. The field of view (FOV) size was 420 μm by 420 μm. The average power delivered to the brain was less than 110 mW. After each recording, the focal plane and imaging position was checked and manually realigned with the initial image if necessary.

### Tests for sociability and social novelty preference

The methods were adapted from established procedures for evaluating social interaction (*36*, *52*). The social test apparatus was a rectangular, two-chambered box (*15*). Each chamber was 28 cm (*L*) by 24 cm (*W*) by 22 cm (*H*). The dividing walls were made from Plexiglas and contained small openings (3.5 cm in diameter) that allowed access to each chamber. The chambers of the social test apparatus were cleaned between trials.

During the habituation period, the test mouse was first placed in the middle chamber and allowed to explore freely for 5 min. Then, in the sociability stage, an unfamiliar male mouse (stranger 1) that had no prior contact with the subject mice was placed in one of the side chambers. The location of stranger 1 in the left or right side chamber was systematically alternated between mice. The stranger mouse was enclosed in a small round wire cage, which allowed nose contact between the bars but prevented fighting. The cage was 11 cm in height with a bottom diameter of 10.5 cm, and bars were spaced 1 cm apart. A weighted cup was placed on the top of the cage to prevent climbing by the test mice. The animals serving as strangers were male mice that had previously been habituated to placement in the small cage. The subject mice were allowed to explore the entire social test apparatus for a 5-min session. The amount of time spent directly exploring each chamber and the number of entries into each chamber were measured by a video camera suspended 1.5 m above the apparatus. Mouth-to-mouth contact was considered direct exploration. In the social novelty test stage, each mouse underwent a second 5-min session to quantitate social preference for a new stranger mouse. A second unfamiliar mouse was placed in the chamber that contained a toy mouse during the sociability session. This second stranger was also enclosed in an identical small wire cage. The test mouse was able to choose to explore the first, already-investigated unfamiliar mouse (old) and the novel unfamiliar mouse (new). As described above, the amount of time spent directly exploring each chamber and the number of transitions between chambers of the apparatus during the social novelty test session were measured. The time spent directly exploring each chamber was recorded, and the differences between groups and within groups were analyzed as previously described (*53*, *54*).

### Activity-dependent chemogenetic experiments

For activity-dependent chemogenetics experiments, 500 nl (each side) of a mixture of two viruses, including 1:2 ratio of rAAV-c-fos-tTA-WPRE-pA and rAAV-TRE3G-DIO-hM4D(Gi)-mCherry-WPRE-pA or rAAV-c-fos-tTA-WPRE-pA and rAAV-TRE3G-DIO-mCherry-WPRE-pA as controls (for details, see table S2), was bilaterally injected to the PrL cortex of the mice (+2.80 AP, ±0.33 ML, +0.45 DV). Then, mice were Dox-fed (1 g/liter) in home cages with the conspecific counterpart. Twenty-five days after injection, the animals were habituated to the experimental environment for 3 days, handled for 3 days, and habituated to intraperitoneal injection by daily saline injections for 3 days. Four weeks after injection, Dox was withdrawn. Thirty-six hours later, before the administration of CNO, the animals underwent the two-chamber social exploration paradigm in which they interacted with familiar and strange male conspecifics. To test whether neural populations activated during the social interaction controlled the social preference and social novelty preference of the animals, we chemogenetically suppressed activation-dependent labeled cells during the two-chamber social assay. The mice were administered with CNO (4 mg/kg) by intraperitoneal injection 30 min followed by the sociability and social novelty preference tests. Animals in the control group, which had been injected with CNO 30 min earlier, were subjected to the two-chamber test in the same apparatus.

### Histology

The mice were deeply anesthetized (sodium pentobarbital; overdose) and then transcardially perfused with saline followed by 4% paraformaldehyde [PFA; in 0.01 M phosphate-bufferred saline (PBS), pH 7.4]. The brains were removed and fixed in 4% PFA overnight at 4°C and then transferred to 15 and 30% sucrose solutions (Sigma-Aldrich, 57-50-1) (48 hours at 4°C). Serial coronal sections (25 μm) were prepared with a vibratome. Brain sections were washed in PBST (PBS, 1% Triton X-100) three times for 10 min, blocked for 1.5 hours in blocking buffer (PBST, 5% normal goat serum), and incubated in primary antibody in blocking buffer overnight at 4°C. On the next day, brains were washed with PBS for 15 min followed by incubation with secondary antibody in blocking buffer at room temperature (RT) for 2 hours. After three washes for 5 min each with PBS, the slices were mounted with fluorescent mounting medium DAPI (4′,6-diamidino-2-phenylindole) (ZSGB-BIO, ZLI-9557). The detailed information of the primary and secondary antibodies was listed in table S2. The injection sites were further confirmed by fluorescence signals. Fluorescence images were then obtained with an Olympus FV-1200 confocal laser scanning microscope (Tokyo, Japan). Images were analyzed with ImageJ (National Institutes of Health, USA).

### Western blotting

NIH-3T3 and HEK293T cells were lysed in a buffer containing 50 mM tris (pH 7.5), 150 mM NaCl, 10 mM MgCl_2_, 1% Triton X-100, 10% glycerol, 1 mM EDTA, 1 mM phenylmethylsulfonyl fluoride, and 1% protease inhibitors. The lysates were centrifuged at 12,000 rpm and 4°C for 15 min. The protein supernatants were boiled in loading buffer for 5 min, electrophoresed on SDS–polyacrylamide gel electrophoresis gels, and transferred onto Millipore polyvinylidene fluoride membranes (Darmstadt, Germany). The membranes were blocked in PBST containing 5% fat-free milk (w/v) for 1 hour at RT, incubated with the appropriate antibody (MeCP2, 1:2000; β-actin, 1:4000) (for details, see table S2) at 4°C overnight, and then incubated with secondary antibody conjugated to horseradish peroxidase for 2 hours at RT. The membranes were stained with standard ECL reagents purchased from Applygen Technologies (Beijing, China) and then imaged by x-ray.

### Mouse direct exploration activity identification

We tracked the movement trajectories of mice using an automatic detecting method and then identified each direct exploration activity (E = cage, mouse, object, new, old) according to the distance between the mouse carrying the miniature microscope and social partner or specific test object. All detected social behaviors were manually checked in this study.

In detail, considering the fiber interference of the mTPM in freely behaving mice, we used a two-step algorithm that combined coarse recognition of the mouse and fine recognition of the head pose. In the first step, the mice bounding box and the centroid coordinates of the mouse’s body in each image frame of the behavioral video were obtained by yolo-v3 (*55*). Then, the mouse head pose coordinates were predicted through cropped image frames by DeepLabCut (*56*). We performed these analyses in the Python and MATLAB (MathWorks) programming environments (for details, see table S3).

To reduce fine spatial fluctuations in the computational determination of the mouse’s locomotor trajectory, we then performed a smooth median filter on the motion of head coordinates. Using the computationally determined trajectories of mouse head movement, we assessed sociability by Euclidean distance between the cage and the head. Moreover, for each image frame, the position, the velocity vector, the total social time, and the social frequency of the mouse were estimated in the arena. The above results were further confirmed by the naked eye. In general, the estimated trajectory matched the mouse’s apparent trajectory in the raw video well.

### Image processing and soma identification

The behavioral video and the corresponding calcium images were captured simultaneously. The frame rate of the neuronal video was 9.76 Hz, while that of the behavioral video was 30 Hz. We resampled and aligned the mouse behavioral video according to the timestamp of the neuronal video, with the error of alignment ∆*t* = 10.33 ± 6.52 ms (mean ± SD).

For each two-photon calcium imaging session, we applied a preprocessing pipeline for image processing using custom software in MATLAB (MathWorks Inc.) (for details, see table S3). First, we performed piecewise rigid motion correction NoRMCorre (*57*) to correct the slight displacement of the FOV caused by mouse movement. Next, we developed a neuron identification framework to effectively extract neuronal soma from the fluorescence video with details as follows: (i) A feature video was constructed by normalized power enhancement of the imaging video to amplify the difference between calcium activity and background noise. (ii) The tubular detection algorithm (*58*) and adaptive threshold segmentation were used on the feature video to obtain the suspected neuron regions in the feature video. (iii) Neuron regions were identified on the basis of signal-to-noise ratio and local spatiotemporal consistency, and the results were manually verified by a self-developed auxiliary tool.

Last, we estimated the displacement fields of the mean images of imaging videos from the same mouse on different days by the “Register virtual stack slices” plugin in Fiji (*59*). The matching template was the mean image of the videos acquired on the first day of the experiment. The same neurons on different days were tracked on the basis of the displacement field and the judging rule; the centroid distance of the same neurons was less than 3 μm, and the overlap rate between the same neurons was greater than 0.75.

### Calcium signal extraction

Subsequently, calcium signals were extracted from identified neurons using the widely used annular ring subtraction (ARS) algorithm (*34*). In detail, the calcium signal Δ*F*/*F* of each neuron was calculated as (i) *F*_ROI_ = *F*_raw_ − *F*_b_, where *F*_ROI_ is the intensity trace subtracted by the background of the video, *F*_raw_ indicates the average intensity curve over the region of interest (ROI) of a cell body, and *F*_b_ is the baseline fluorescence of the background, which was obtained by concatenating the minimum values of the image stack; (ii) *F*_con_ = *F*_ring_ − *F*_b_, where *F*_con_ is the intensity trace of *z*-axis contamination, and *F*_ring_ denotes the raw intensity trace of a ring area around the cell; (iii) *F*_sig_ = *F*_ROI_ − α × *F*_con_, where *F*_sig_ stands for the actual intensity change of a neuron after subtracting the background drift and *z*-axis contamination, α indicates the degree of contamination, and α = 1 in this paper; and (iv) Δ*F*/*F* = *F*_sig_/*F*_0_, where *F*_0_ is the baseline value of *F*_ROI_, and *F*_0_ is estimated by the mean value of *F*_ROI_.

### Subgrouping of TNs, PNs, and INs into functional ensembles

To identify the key functional participants in the modulation of social behavior, we divided each of TNs, PNs, and INs into two functional subgroups named SANs and SINs according to their correlations with the social behavior (*60*). The classification algorithm involves three steps: (i) The similarity *S_i_* between the calcium signal of *i*th neuron *C_i_* (Δ*F*/*F*) and mouse social behavior vector *B*_M_ was first defined as *B*_M_ · *C_i_*/(∣*B*_M_∣^2^ + ∣*C_i_*∣^2^), where the binary behavior *B*_M_ could only be 0 and 1, with 0 standing for the free-moving state of the mouse wearing an mTPM and 1 standing for the social interaction between the observed mouse and the caged mouse. (ii) Next, for each neuron, we randomly permuted *B*_M_ *k* times, and then similarity values $S_{i}^{k}(k=1,2,\ldots )$were obtained according to the definition described in (i), which constituted a null distribution assuming that the calcium signals were not correlated with social behavior (*k* = 1000 in this paper). (iii) A neuron was classified as a SAN if its *S_i_* was greater than the 99.17% percentile of $S_{i}^{k}$ or as a SIN if its *S_i_* was lower than the 0.83% percentile of $S_{i}^{k}$. Neurons that did not meet either of the two conditions above were classified as Others (other neurons).

### Quantification of the signal-behavior correlation

To quantify the degree of relevance of TNs, PNs, and INs with social behaviors, we evaluated the signal-behavior correlation between the calcium signal *C_i_* and the social behavior vector *B*_M_. In detail, for SANs and SINs, as defined above, after the similarity *S_i_* between its calcium signal *C_i_* (Δ*F*/*F*) and the mouse social behavior vector B_*S*1_ was obtained, we extracted the similarities of SAN and SIN ensembles and calculated their mean values as the signal-behavior correlations of the SAN and SIN ensembles in each mouse.

### Calculation of the event average Δ*F*/*F* calcium traces

To investigate the typical pattern of calcium responses of SAN and SIN functional subpopulations of neurons during specific exploration behaviors, we defined the event average Δ*F*/*F* calcium trace, which represents the average calcium response of a neuronal assembly of a mouse at the beginning or end of all specific exploration events. Concretely speaking, for the beginning of any exploration event E (E = mouse, object), we calculated the event average Δ*F*/*F* calcium trace through the following steps: (i) First, all the valid initial fragments of E during experiments were marked. The valid initial fragments indicated time segments of 8 s, the first 4 s of which was during the NE state and the last 4 s of which was during the E state. (ii) The calcium signal Δ*F*/*F* of each neuron was smoothed by a low-pass filter with a span of 5, and all the signal segments that fell into the valid initial fragments of E were aligned and averaged, allowing us to obtain the event E–triggered signal of this neuron. (iii) The event average Δ*F*/*F* calcium trace of a specific neuronal assembly was obtained by averaging all the event E–triggered signals of whole neurons in this assembly. In the same way, when any exploration event E ended, the valid fragments described in (i) were inversed, with the first 4 s representing the E state and the last 4 s representing the NE state. For the event average relative Δ*F*/*F* calcium traces, the baseline value was subtracted from the calcium signal of each neuron before alignment, and the baseline value was estimated by averaging all Δ*F*/*F* values during the NE state.

### Neuron social engagement analysis

To further quantify the degree of engagement of SAN and SIN functional ensemble during social behavior, two parameters were used: the consistency and percentage of neurons (*8*). The consistency of a neuron was the ratio of engaged social events relative to the total number of social events during a trial; the percentage of neurons was the ratio of engaged neurons relative to all neurons in a specific assembly for a single social interaction event. Here, we defined neuronal engagement according to the three-sigma rule, that is, a neuron was engaged in a social event if *n* points of its calcium activity were three times higher than the noise level of the whole signal (*n* = 10 in this paper); we estimated the noise level by filtering the Δ*F*/*F* calcium trace with a 50-order high-pass filter with a cutoff frequency of 1 Hz and then calculated the SD of the filtered trace.

### Global neuronal network analysis of INs, PNs, and TNs

Pairwise functional connectivity is modulated during social states and related to social behavioral choice. We sought to investigate how the functional network constructed by different types of neurons (INs, PNs, and TNs) is engaged during mice’s social stages.

The connectivity matrices were established by the following procedures: Δ*F*/*F* traces were split into blocks based on social vectors, while those segments of interest were subsequently concatenated. In other words, the selected traces were shortened by removing fragments of uninterested states (*35*). Because of the splicing operation, a linear correlation coefficient was more suitable. Given that Pearson’s correlation coefficient infers significant linear correlations between two neurons (*61*), we calculated pairwise Pearson’s correlations between the spliced Δ*F*/*F* traces of all neurons for social states. Graph theory was applied to describe the topology of the neuronal networks using the Brain Connectivity Toolbox (*62*), and the generalized clustering coefficient was introduced to characterize both positive and negative correlations.

### Detection of EANs among INs

To explore the patterns of INs encoding social behaviors, we first identified the neurons associated with various exploration behaviors E in the three-stage paradigm (habituation, sociability, and social novelty preference). Briefly, a neuron was considered an EAN if it exhibited a response to the corresponding exploration event. Here, we defined a response as a significant increase in the mean amplitude of the Δ*F*/*F* calcium signal during E events compared with NE events. (i) Specifically, we calculated the mean amplitude of the *i*th neuron of a mouse during exploration E (*c*_*i*,E_ = < *B*_E_ · *C_i_* >). The bracket represents the mean value of a time sequence, and 1 in *B_C_* represents the exploration of empty cages. (ii) The mean amplitude during NE was calculated in the same way (*c*_*i*,NE_ = < *B*_NE_ · *C_i_* >). (iii) The difference (*c*_*i*,E−NE_ = *c*_*i*,E_ − *c*_*i*,NE_) was measured as the mean increase in the calcium signal amplitude triggered by these events. (iv) The *i*th neuron was categorized as an EAN if it met < *c*_*i*,E−NE_ > β × noise_*i*,NE_, where β is the significance threshold for the ratio of *c*_*i*,E−NE_ to noise_*i*,NE_, and β = 0.5 in this paper. Here, noise_*i*,NE_ represents the noise level of the *i*th neuron during the NE state. To estimate noise_*i*,NE_, we first separated the signal segments when the observed mouse was in the NE state and then connected them to form a new signal. Then, we filtered the new Δ*F*/*F* calcium trace with a 50-order high-pass filter with a cutoff frequency of 1 Hz and calculated the SD of the filtered trace.

### Detection of social cue–preferred neurons among INs

To further explore the deficits of *MECP2*-TG mice under social behaviors, we sought to identify functional ensembles of INs that had a preferred response to new and old social behaviors. In this paper, we identified NewPNs and OldPNs. These two neuronal types among INs are collectively called social cue–preferred neurons.

More specifically, a neuron was classified as a NewPN if it met the following three criteria: (i) It was not classified as a CageN during the habituation stage, (ii) it was not classified as either an ON or MN during the sociability stage, and (iii) it was not classified as an OldN but was classified as a NewN during the social novelty stage; that is, as the expression in set theory

{neuron i of NewPN ∣ i∈NewN,i∉CageN ∪ ON ∪ MN ∪ OldN}

and an OldPN was defined as

{neuron i of OldPN ∣ i∈OldN,i∉CageN ∪ ON ∪ MN ∪ NewN}

### Average behavioral correlation matrix of EANs and social cue–preferred INs

The average correlation matrix of single-neuron responses across different behavioral stages can be used to detect similar performance when neurons code different tasks (*61*, *63*). As mentioned above, on the basis of the average Δ*F*/*F* of each calcium trace, the behavioral correlation matrix of each neuron was obtained by calculating the Pearson’s correlation coefficients of Δ*F*/*F* segments during different states. Last, the average behavioral correlation matrix was obtained by averaging the behavioral correlation matrix of different categories of EANs and social cue–preferred INs.

### Analysis of the Ca^2+^ dynamics of social cue–preferred INs in socially activated epochs

The social cue–preferred INs in the PrL area were not activated in every social epoch, which inspired us to focus on the kinetic characteristics of socially activated epochs. Considering the classic rapid rise and slow decline of calcium signal dynamics, we defined the socially activated epochs as epochs involved socially triggered calcium signal peaks in 4 s after the start of the social epoch. The socially triggered peak was referred to as the maximum value of the calcium signal in the 4 s before and after the start of the social epoch. To prevent cross-talk from adjacent social events, social epochs with social or presocial exploration times shorter than 4 s were discarded, and the rest of the social epochs were regarded as socially nonactivated epochs.

We estimated the activation probability (*P*_up_) of each social cue–preferred IN, by dividing the number of socially activated epochs by the sum of socially activated and nonactivated epochs. Then, for socially activated epochs, we characterized calcium dynamic activity by measuring the rise time (Time to peak_up_) and the extent of the increase (Amplitude_up_). The rise time was defined as the time from the beginning of the social epoch to the appearance of the socially triggered peak. The extent of the increase was defined as the difference in Δ*F*/*F* between the socially triggered peak and baseline. The baseline level was the minimum value of the calcium signal 4 s before the start of the social epoch. The kinetic features of all socially activated events for the same neuron were finally averaged as its representative value.

### Quantification and statistical analysis

All data are shown as the mean ± SEM unless otherwise specified. All box and whisker plots show the median, 25th to 75th percentile (box), and min and max (whiskers) of the data. All statistical analyses were conducted using GraphPad Prism (v8.0.2) or custom routines in MATLAB (Mathworks) and are described in the respective figure legends. Where applicable, outliers were identified using the ROUT (robust regression and outlier removal) method. Assumptions of normality were tested with the D’Agostino-Pearson normality test. All two-group comparisons were calculated using Student’s *t* test (two-tailed, paired, or unpaired), when data followed a Gaussian distribution. Otherwise, we used the Wilcoxon matched-pairs signed rank test for paired data and used nonparametric Mann-Whitney *U* test for unpaired data. For comparisons of multiple groups, either analysis of variance (ANOVA) or the Kruskal-Wallis test was performed as appropriate. Post hoc analysis was performed using Dunn’s multiple comparisons test or Bonferroni’s multiple comparisons test where appropriate. The results were considered statistically significant when *P* < 0.05. Details of the statistical analyses are provided in table S1.

## Notes

View/request a protocol for this paper from *Bio-protocol*.

## References

[1] C. Chevallier, G. Kohls, V. Troiani, E. S. Brodkin, R. T. Schultz, The social motivation theory of autism. Trends Cogn. Sci. 16, 231–239 (2012). 22425667 10.1016/j.tics.2012.02.007 PMC3329932

[2] M. F. Green, W. P. Horan, J. Lee, Social cognition in schizophrenia. Nat. Rev. Neurosci. 16, 620–631 (2015). 26373471 10.1038/nrn4005

[3] A. Kupferberg, L. Bicks, G. Hasler, Social functioning in major depressive disorder. Neurosci. Biobehav. Rev. 69, 313–332 (2016). 27395342 10.1016/j.neubiorev.2016.07.002

[4] L. K. Bicks, H. Koike, S. Akbarian, H. Morishita, Prefrontal cortex and social cognition in mouse and man. Front. Psychol. 6, 1805 (2015). 26635701 10.3389/fpsyg.2015.01805 PMC4659895

[5] R. Báez-Mendoza, E. P. Mastrobattista, A. J. Wang, Z. M. Williams, Social agent identity cells in the prefrontal cortex of interacting groups of primates. Science 374, eabb4149 (2021). 34672743 10.1126/science.abb4149 PMC8571805

[6] L. Kingsbury, S. Huang, T. Raam, L. S. Ye, D. Wei, R. K. Hu, L. Ye, W. Hong, Cortical representations of conspecific sex shape social behavior. Neuron 107, 941–953.e7 (2020). 32663438 10.1016/j.neuron.2020.06.020 PMC7486272

[7] F. Krueger, A. K. Barbey, J. Grafman, The medial prefrontal cortex mediates social event knowledge. Trends Cogn. Sci. 13, 103–109 (2009). 19223228 10.1016/j.tics.2008.12.005

[8] B. Liang, L. Zhang, G. Barbera, W. Fang, J. Zhang, X. Chen, R. Chen, Y. Li, D. T. Lin, Distinct and dynamic on and off neural ensembles in the prefrontal cortex code social exploration. Neuron 100, 700–714.e9 (2018). 30269987 10.1016/j.neuron.2018.08.043 PMC6224317

[9] A. Noritake, T. Ninomiya, M. Isoda, Social reward monitoring and valuation in the macaque brain. Nat. Neurosci. 21, 1452–1462 (2018). 30224807 10.1038/s41593-018-0229-7

[10] H. Xu, L. Liu, Y. Tian, J. Wang, J. Li, J. Zheng, H. Zhao, M. He, T. L. Xu, S. Duan, H. Xu, A disinhibitory microcircuit mediates conditioned social fear in the prefrontal cortex. Neuron 102, 668–682.e5 (2019). 30898376 10.1016/j.neuron.2019.02.026

[11] O. Yizhar, L. E. Fenno, M. Prigge, F. Schneider, T. J. Davidson, D. J. O’Shea, V. S. Sohal, I. Goshen, J. Finkelstein, J. T. Paz, K. Stehfest, R. Fudim, C. Ramakrishnan, J. R. Huguenard, P. Hegemann, K. Deisseroth, Neocortical excitation/inhibition balance in information processing and social dysfunction. Nature 477, 171–178 (2011). 21796121 10.1038/nature10360 PMC4155501

[12] A. C. Brumback, I. T. Ellwood, C. Kjaerby, J. Iafrati, S. Robinson, A. T. Lee, T. Patel, S. Nagaraj, F. Davatolhagh, V. S. Sohal, Identifying specific prefrontal neurons that contribute to autism-associated abnormalities in physiology and social behavior. Mol. Psychiatry 23, 2078–2089 (2018). 29112191 10.1038/mp.2017.213 PMC6594833

[13] Y. Kim, K. U. Venkataraju, K. Pradhan, C. Mende, J. Taranda, S. C. Turaga, I. Arganda-Carreras, L. Ng, M. J. Hawrylycz, K. S. Rockland, H. S. Seung, P. Osten, Mapping social behavior-induced brain activation at cellular resolution in the mouse. Cell Rep. 10, 292–305 (2015). 25558063 10.1016/j.celrep.2014.12.014 PMC4294964

[14] E. Lee, I. Rhim, J. W. Lee, J. W. Ghim, S. Lee, E. Kim, M. W. Jung, Enhanced neuronal activity in the medial prefrontal cortex during social approach behavior. J. Neurosci. 36, 6926–6936 (2016). 27358451 10.1523/JNEUROSCI.0307-16.2016 PMC6604896

[15] M. Murugan, H. J. Jang, M. Park, E. M. Miller, J. Cox, J. P. Taliaferro, N. F. Parker, V. Bhave, H. Hur, Y. Liang, A. R. Nectow, J. W. Pillow, I. B. Witten, Combined social and spatial coding in a descending projection from the prefrontal cortex. Cell 171, 1663–1677.e16 (2017). 29224779 10.1016/j.cell.2017.11.002 PMC5889923

[16] L. Pinto, Y. Dan, Cell-type-specific activity in prefrontal cortex during goal-directed behavior. Neuron 87, 437–450 (2015). 26143660 10.1016/j.neuron.2015.06.021 PMC4506259

[17] M. Rigotti, O. Barak, M. R. Warden, X. J. Wang, N. D. Daw, E. K. Miller, S. Fusi, The importance of mixed selectivity in complex cognitive tasks. Nature 497, 585–590 (2013). 23685452 10.1038/nature12160 PMC4412347

[18] M. Shafi, Y. Zhou, J. Quintana, C. Chow, J. Fuster, M. Bodner, Variability in neuronal activity in primate cortex during working memory tasks. Neuroscience 146, 1082–1108 (2007). 17418956 10.1016/j.neuroscience.2006.12.072

[19] W. Cao, S. Lin, Q. Q. Xia, Y. L. Du, Q. Yang, M. Y. Zhang, Y. Q. Lu, J. Xu, S. M. Duan, J. Xia, G. Feng, J. Xu, J. H. Luo, Gamma oscillation dysfunction in mPFC leads to social deficits in neuroligin 3 R451C knockin mice. Neuron 97, 1253–1260.e7 (2018). 29503190 10.1016/j.neuron.2018.02.001

[20] L. Liu, H. Xu, J. Wang, J. Li, Y. Tian, J. Zheng, M. He, T. L. Xu, Z. Y. Wu, X. M. Li, S. M. Duan, H. Xu, Cell type-differential modulation of prefrontal cortical GABAergic interneurons on low gamma rhythm and social interaction. Sci. Adv. 6, eaay4073 (2020). 32832654 10.1126/sciadv.aay4073 PMC7439507

[21] Q. Sun, X. Li, A. Li, J. Zhang, Z. Ding, H. Gong, Q. Luo, Ventral hippocampal-prefrontal interaction affects social behavior via parvalbumin positive neurons in the medial prefrontal cortex. iScience 23, 100894 (2020). 32092698 10.1016/j.isci.2020.100894 PMC7038035

[22] C. Zhang, H. Zhu, Z. Ni, Q. Xin, T. Zhou, R. Wu, G. Gao, Z. Gao, H. Ma, H. Li, M. He, J. Zhang, H. Cheng, H. Hu, Dynamics of a disinhibitory prefrontal microcircuit in controlling social competition. Neuron 110, 516–531.e6 (2021). 34793692 10.1016/j.neuron.2021.10.034

[23] A. L. Collins, J. M. Levenson, A. P. Vilaythong, R. Richman, D. L. Armstrong, J. L. Noebels, J. D. Sweatt, H. Y. Zoghbi, Mild overexpression of MeCP2 causes a progressive neurological disorder in mice. Hum. Mol. Genet. 13, 2679–2689 (2004). 15351775 10.1093/hmg/ddh282

[24] R. C. Samaco, C. Mandel-Brehm, C. M. McGraw, C. A. Shaw, B. E. McGill, H. Y. Zoghbi, Crh and Oprm1 mediate anxiety-related behavior and social approach in a mouse model of MECP2 duplication syndrome. Nat. Genet. 44, 206–211 (2012). 22231481 10.1038/ng.1066 PMC3267865

[25] B. Yu, B. Yuan, J. K. Dai, T. L. Cheng, S. N. Xia, L. J. He, Y. T. Yuan, Y. F. Zhang, H. T. Xu, F. Q. Xu, Z. F. Liang, Z. L. Qiu, Reversal of social recognition deficit in adult mice with MECP2 duplication via normalization of MeCP2 in the medial prefrontal cortex. Neurosci. Bull. 36, 570–584 (2020). 32144612 10.1007/s12264-020-00467-w PMC7271088

[26] E. S. Na, E. D. Nelson, M. Adachi, A. E. Autry, M. A. Mahgoub, E. T. Kavalali, L. M. Monteggia, A mouse model for MeCP2 duplication syndrome: MeCP2 overexpression impairs learning and memory and synaptic transmission. J. Neurosci. 32, 3109–3117 (2012). 22378884 10.1523/JNEUROSCI.6000-11.2012 PMC3835557

[27] L. Sun, R. Chen, L. Li, B. Yuan, K. Song, N. Pan, T.-L. Cheng, S. Chang, K. Lin, X. He, Q. Wu, F. Xu, Z. Qiu, X. Wang, Visualization and correction of social abnormalities-associated neural ensembles in adult MECP2 duplication mice. Sci. Bull. 65, 1192–1202 (2020). 10.1016/j.scib.2020.03.026 36659149

[28] F. Babiloni, F. Cincotti, D. Mattia, M. Mattiocco, F. De Vico Fallani, A. Tocci, L. Bianchi, M. G. Marciani, L. Astolfi, Hypermethods for EEG hyperscanning. Conf. Proc. IEEE Eng. Med. Biol. Soc. 2006, 3666–3669 (2006). 17945788 10.1109/IEMBS.2006.260754

[29] L. Kingsbury, S. Huang, J. Wang, K. Gu, P. Golshani, Y. E. Wu, W. Hong, Correlated neural activity and encoding of behavior across brains of socially interacting animals. Cell 178, 429–446.e16 (2019). 31230711 10.1016/j.cell.2019.05.022 PMC6625832

[30] P. R. Montague, G. S. Berns, J. D. Cohen, S. M. McClure, G. Pagnoni, M. Dhamala, M. C. Wiest, I. Karpov, R. D. King, N. Apple, R. E. Fisher, Hyperscanning: Simultaneous fMRI during linked social interactions. Neuroimage 16, 1159–1164 (2002). 12202103 10.1006/nimg.2002.1150

[31] W. Zong, R. Wu, S. Chen, J. Wu, H. Wang, Z. Zhao, G. Chen, R. Tu, D. Wu, Y. Hu, Y. Xu, Y. Wang, Z. Duan, H. Wu, Y. Zhang, J. Zhang, A. Wang, L. Chen, H. Cheng, Miniature two-photon microscopy for enlarged field-of-view, multi-plane and long-term brain imaging. Nat. Methods 18, 46–49 (2021). 33408404 10.1038/s41592-020-01024-z

[32] W. Zong, R. Wu, M. Li, Y. Hu, Y. Li, J. Li, H. Rong, H. Wu, Y. Xu, Y. Lu, H. Jia, M. Fan, Z. Zhou, Y. Zhang, A. Wang, L. Chen, H. Cheng, Fast high-resolution miniature two-photon microscopy for brain imaging in freely behaving mice. Nat. Methods 14, 713–719 (2017). 28553965 10.1038/nmeth.4305

[33] A. Lin, R. Adolphs, A. Rangel, Social and monetary reward learning engage overlapping neural substrates. Soc. Cogn. Affect. Neurosci. 7, 274–281 (2012). 21427193 10.1093/scan/nsr006 PMC3304477

[34] T. W. Chen, T. J. Wardill, Y. Sun, S. R. Pulver, S. L. Renninger, A. Baohan, E. R. Schreiter, R. A. Kerr, M. B. Orger, V. Jayaraman, L. L. Looger, K. Svoboda, D. S. Kim, Ultrasensitive fluorescent proteins for imaging neuronal activity. Nature 499, 295–300 (2013). 23868258 10.1038/nature12354 PMC3777791

[35] H. Cao, M. M. Plichta, A. Schäfer, L. Haddad, O. Grimm, M. Schneider, C. Esslinger, P. Kirsch, A. Meyer-Lindenberg, H. Tost, Test-retest reliability of fMRI-based graph theoretical properties during working memory, emotion processing, and resting state. Neuroimage 84, 888–900 (2014). 24055506 10.1016/j.neuroimage.2013.09.013

[36] S. S. Moy, J. J. Nadler, A. Perez, R. P. Barbaro, J. M. Johns, T. R. Magnuson, J. Piven, J. N. Crawley, Sociability and preference for social novelty in five inbred strains: An approach to assess autistic-like behavior in mice. Genes Brain Behav. 3, 287–302 (2004). 15344922 10.1111/j.1601-1848.2004.00076.x

[37] J. R. Kurian, M. E. Bychowski, R. M. Forbes-Lorman, C. J. Auger, A. P. Auger, Mecp2 organizes juvenile social behavior in a sex-specific manner. J. Neurosci. 28, 7137–7142 (2008). 18614683 10.1523/JNEUROSCI.1345-08.2008 PMC2569867

[38] H. Liu, Z. Qiu, Overexpression of MECP2 in the suprachiasmatic nucleus alters circadian rhythm and induces abnormal social behaviors. Neurosci. Bull. 37, 1713–1717 (2021). 34283398 10.1007/s12264-021-00746-0 PMC8643386

[39] Y. Sztainberg, H.-m. Chen, J. W. Swann, S. Hao, B. Tang, Z. Wu, J. Tang, Y.-W. Wan, Z. Liu, F. Rigo, H. Y. Zoghbi, Reversal of phenotypes in MECP2 duplication mice using genetic rescue or antisense oligonucleotides. Nature 528, 123–126 (2015). 26605526 10.1038/nature16159 PMC4839300

[40] R. J. Platt, S. Chen, Y. Zhou, M. J. Yim, L. Swiech, H. R. Kempton, J. E. Dahlman, O. Parnas, T. M. Eisenhaure, M. Jovanovic, D. B. Graham, S. Jhunjhunwala, M. Heidenreich, R. J. Xavier, R. Langer, D. G. Anderson, N. Hacohen, A. Regev, G. Feng, P. A. Sharp, F. Zhang, CRISPR-Cas9 knockin mice for genome editing and cancer modeling. Cell 159, 440–455 (2014). 25263330 10.1016/j.cell.2014.09.014 PMC4265475

[41] D. Wei, V. Talwar, D. Lin, Neural circuits of social behaviors: Innate yet flexible. Neuron 109, 1600–1620 (2021). 33705708 10.1016/j.neuron.2021.02.012 PMC8141016

[42] J. L. Silverman, M. Yang, C. Lord, J. N. Crawley, Behavioural phenotyping assays for mouse models of autism. Nat. Rev. Neurosci. 11, 490–502 (2010). 20559336 10.1038/nrn2851 PMC3087436

[43] W. Yu, Y. C. Yen, Y. H. Lee, S. Tan, Y. Xiao, H. Lokman, A. K. T. Ting, H. Ganegala, T. Kwon, W. K. Ho, H. S. Je, Prenatal selective serotonin reuptake inhibitor (SSRI) exposure induces working memory and social recognition deficits by disrupting inhibitory synaptic networks in male mice. Mol. Brain 12, 29 (2019). 30935412 10.1186/s13041-019-0452-5 PMC6444596

[44] L. A. Gunaydin, L. Grosenick, J. C. Finkelstein, I. V. Kauvar, L. E. Fenno, A. Adhikari, S. Lammel, J. J. Mirzabekov, R. D. Airan, K. A. Zalocusky, K. M. Tye, P. Anikeeva, R. C. Malenka, K. Deisseroth, Natural neural projection dynamics underlying social behavior. Cell 157, 1535–1551 (2014). 24949967 10.1016/j.cell.2014.05.017 PMC4123133

[45] Y. Li, A. Mathis, B. F. Grewe, J. A. Osterhout, B. Ahanonu, M. J. Schnitzer, V. N. Murthy, C. Dulac, Neuronal representation of social information in the medial amygdala of awake behaving mice. Cell 171, 1176–1190.e17 (2017). 29107332 10.1016/j.cell.2017.10.015 PMC5731476

[46] R. Hattori, K. V. Kuchibhotla, R. C. Froemke, T. Komiyama, Functions and dysfunctions of neocortical inhibitory neuron subtypes. Nat. Neurosci. 20, 1199–1208 (2017). 28849791 10.1038/nn.4619 PMC7082034

[47] Q. Sun, X. Li, M. Ren, M. Zhao, Q. Zhong, Y. Ren, P. Luo, H. Ni, X. Zhang, C. Zhang, J. Yuan, A. Li, M. Luo, H. Gong, Q. Luo, A whole-brain map of long-range inputs to GABAergic interneurons in the mouse medial prefrontal cortex. Nat. Neurosci. 22, 1357–1370 (2019). 31285615 10.1038/s41593-019-0429-9

[48] S. Zhang, M. Xu, W.-C. Chang, C. Ma, J. P. Hoang Do, D. Jeong, T. Lei, J. L. Fan, Y. Dan, Organization of long-range inputs and outputs of frontal cortex for top-down control. Nat. Neurosci. 19, 1733–1742 (2016). 27749828 10.1038/nn.4417 PMC5127741

[49] G. Dölen, A. Darvishzadeh, K. W. Huang, R. C. Malenka, Social reward requires coordinated activity of nucleus accumbens oxytocin and serotonin. Nature 501, 179–184 (2013). 24025838 10.1038/nature12518 PMC4091761

[50] M. L. Donegan, F. Stefanini, T. Meira, J. A. Gordon, S. Fusi, S. A. Siegelbaum, Coding of social novelty in the hippocampal CA2 region and its disruption and rescue in a 22q11.2 microdeletion mouse model. Nat. Neurosci. 23, 1365–1375 (2020). 33077947 10.1038/s41593-020-00720-5 PMC8861630

[51] H.-T. Chao, H. Chen, R. C. Samaco, M. Xue, M. Chahrour, J. Yoo, J. L. Neul, S. Gong, H.-C. Lu, N. Heintz, M. Ekker, J. L. R. Rubenstein, J. L. Noebels, C. Rosenmund, H. Y. Zoghbi, Dysfunction in GABA signalling mediates autism-like stereotypies and Rett syndrome phenotypes. Nature 468, 263–269 (2010). 21068835 10.1038/nature09582 PMC3057962

[52] B. Rein, K. Ma, Z. Yan, A standardized social preference protocol for measuring social deficits in mouse models of autism. Nat. Protoc. 15, 3464–3477 (2020). 32895524 10.1038/s41596-020-0382-9 PMC8103520

[53] S. Nieuwenhuis, B. U. Forstmann, E. J. Wagenmakers, Erroneous analyses of interactions in neuroscience: A problem of significance. Nat. Neurosci. 14, 1105–1107 (2011). 21878926 10.1038/nn.2886

[54] K. R. Nygaard, S. E. Maloney, J. D. Dougherty, Erroneous inference based on a lack of preference within one group: Autism, mice, and the social approach task. Autism Res. 12, 1171–1183 (2019). 31187603 10.1002/aur.2154 PMC6688965

[55] A. Farhadi, J. Redmon, in *Computer Vision and Pattern Recognition* (Springer Berlin/Heidelberg, 2018), pp. 1804–2767.

[56] A. Mathis, P. Mamidanna, K. M. Cury, T. Abe, V. N. Murthy, M. W. Mathis, M. Bethge, DeepLabCut: Markerless pose estimation of user-defined body parts with deep learning. Nat. Neurosci. 21, 1281–1289 (2018). 30127430 10.1038/s41593-018-0209-y

[57] E. A. Pnevmatikakis, A. Giovannucci, NoRMCorre: An online algorithm for piecewise rigid motion correction of calcium imaging data. J. Neurosci. Methods 291, 83–94 (2017). 28782629 10.1016/j.jneumeth.2017.07.031

[58] A. Vasilevskiy, K. Siddiqi, Flux maximizing geometric flows. IEEE Trans. Pattern Anal. Mach. Intell. 24, 1565–1578 (2002).

[59] J. Schindelin, I. Arganda-Carreras, E. Frise, V. Kaynig, M. Longair, T. Pietzsch, S. Preibisch, C. Rueden, S. Saalfeld, B. Schmid, J.-Y. Tinevez, D. J. White, V. Hartenstein, K. Eliceiri, P. Tomancak, A. Cardona, Fiji: An open-source platform for biological-image analysis. Nat. Methods 9, 676–682 (2012). 22743772 10.1038/nmeth.2019 PMC3855844

[60] J. P. Hamm, D. S. Peterka, J. A. Gogos, R. Yuste, Altered cortical ensembles in mouse models of schizophrenia. Neuron 94, 153–167.e8 (2017). 28384469 10.1016/j.neuron.2017.03.019 PMC5394986

[61] J. Niessing, R. W. Friedrich, Olfactory pattern classification by discrete neuronal network states. Nature 465, 47–52 (2010). 20393466 10.1038/nature08961

[62] M. Rubinov, O. Sporns, Complex network measures of brain connectivity: Uses and interpretations. Neuroimage 52, 1059–1069 (2010). 19819337 10.1016/j.neuroimage.2009.10.003

[63] J. Gründemann, Y. Bitterman, T. Lu, S. Krabbe, B. F. Grewe, M. J. Schnitzer, A. Lüthi, Amygdala ensembles encode behavioral states. Science 364, (2019). 10.1126/science.aav8736 31000636

