# Supplementary material for: Encoding of social novelty by sparse GABAergic neural ensembles in the prelimbic cortex
Source: Sci Adv. 2022 Aug 31;8(35):eabo4884. doi: 10.1126/sciadv.abo4884 (PMC9432833; doi:10.1126/sciadv.abo4884)
Supplement: Supplementary file 1 — Figs. S1 to S14 Tables S1 to S3 [file sciadv.abo4884_sm.pdf]

Science Advances
AAAS

Supplementary Materials for

Encoding of social novelty by sparse GABAergic neural ensembles in the prelimbic cortex

Zhe Zhao *et al.*

Corresponding author: Jue Zhang, zhangjue@pku.edu.cn; Liangyi Chen, lychen@pku.edu.cn; Haitao Wu, wuht@bmi.ac.cn

Sci. Adv. **8**, eabo4884 (2022)
DOI: 10.1126/sciadv.abo4884

The PDF file includes:

Figs. S1 to S14
Tables S1 to S3
Legends for movies S1 to S7

Other Supplementary Material for this manuscript includes the following:

Movies S1 to S7

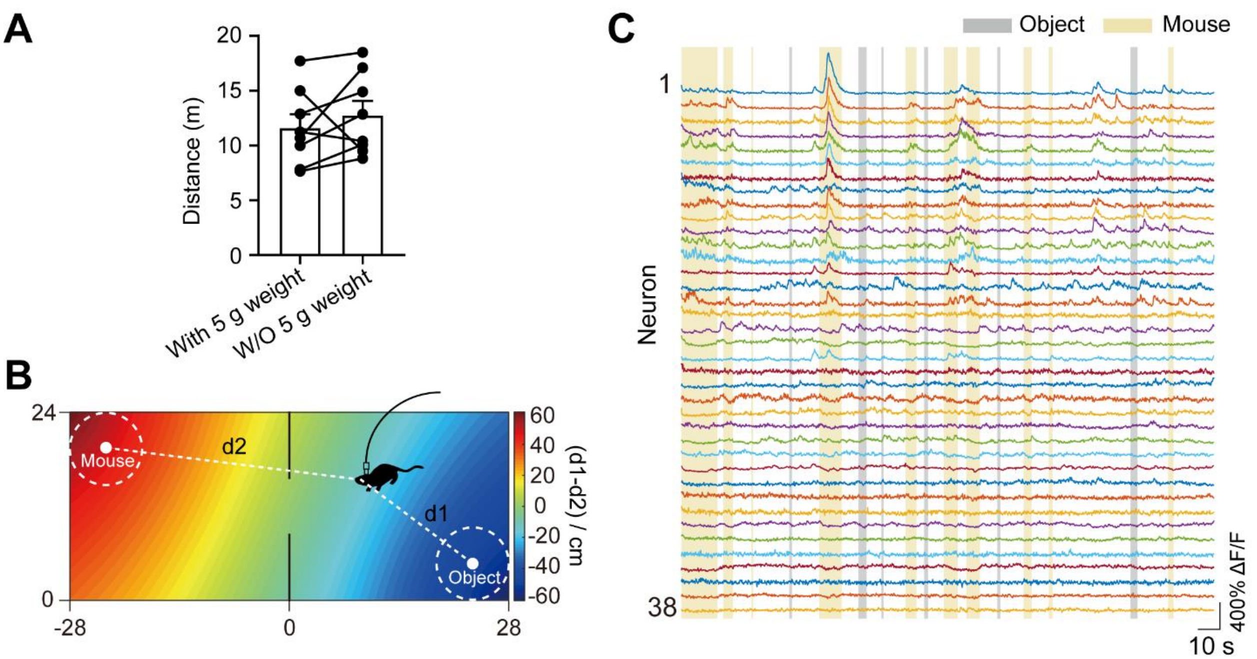

Fig. S1. Plots showing behavior and calcium traces in mice.

(A) The head-mounted microscope did not affect the travel distance of the mice suspended with helium balloons. Two-tailed paired *t* test. The data are shown as the mean ± SEM. ns, no significant difference.

(B) The distribution of d1-d2 values in the two-chamber apparatus, where d1 represents the distance between the mouse and the center of the object cage, and d2 represents that between the mouse and the center of the mouse cage.

(C) Representative calcium traces of 38 neurons from a typical WT mouse. Data is presented for a 5-min period of the recording. Each row represents a neuron in the imaging field. The colored bands reflect the different exploration states of the mouse.

For detailed statistical information, see Table S1.

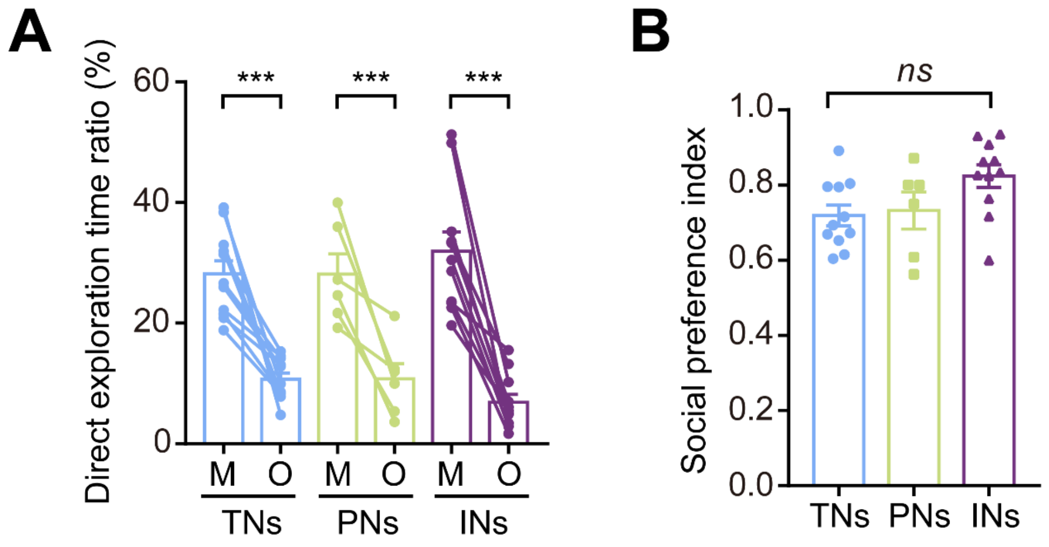

Fig. S2. Statistical analysis of exploration behaviors.

(A) Direct exploration time ratio of the stranger mouse (M) and the object (O) chambers by mice in which TNs, PNs, and INs were imaged. Two-way ANOVA followed by the Bonferroni *post hoc* test. The data are shown as the mean ± SEM. *p < 0.05, **p < 0.01, ***p < 0.001; *ns*, no significance (p > 0.05).

(B) No difference was found in social preference index between mice in which TNs, PNs, and INs were imaged. One-way ANOVA followed by Bonferroni *post hoc* test. The data are shown as the mean ± SEM. *ns*, no significance (p > 0.05).

For detailed statistical information, see Table S1.

Fig. S3

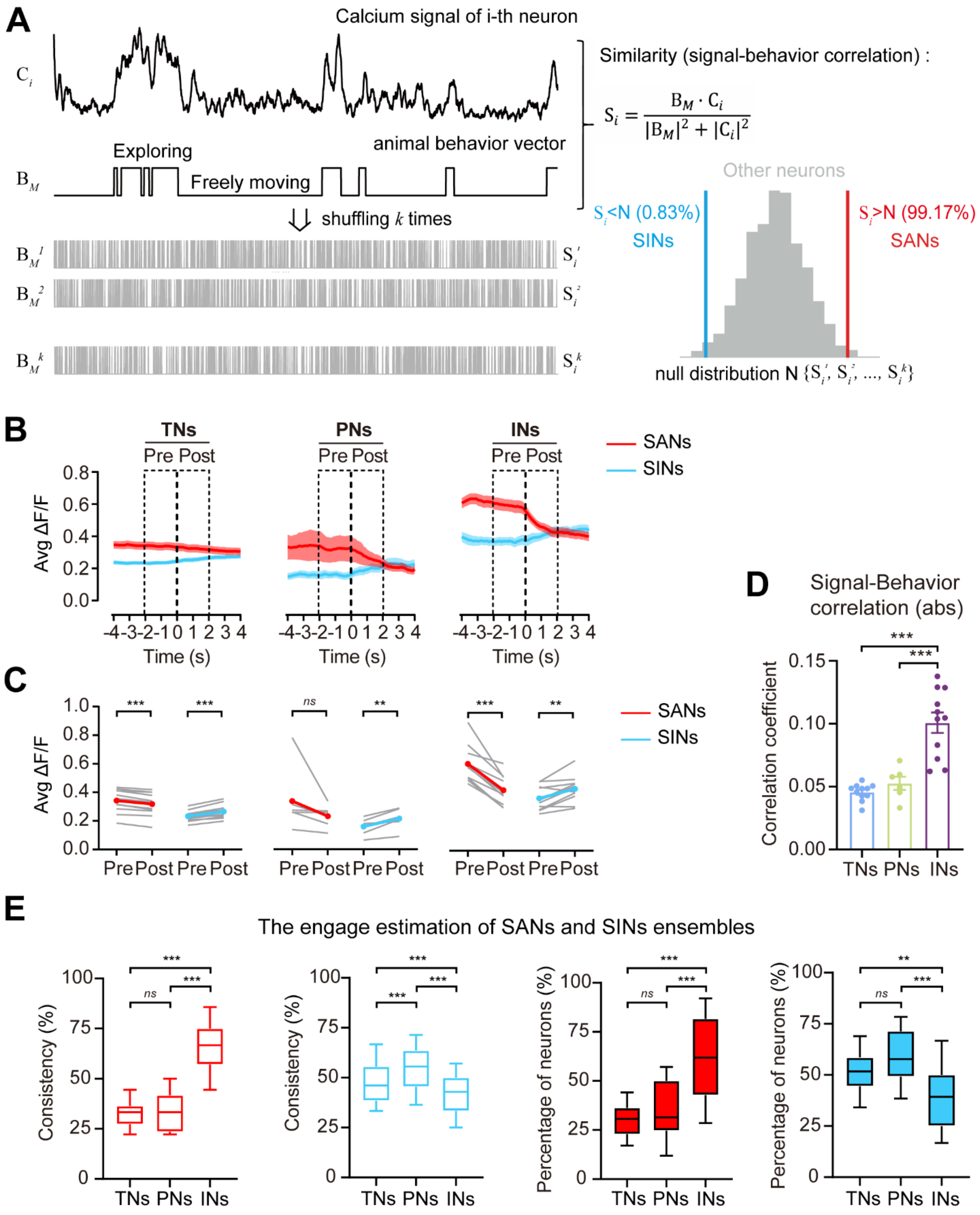

A
Calcium signal of i-th neuron
Similarity (signal-behavior correlation) :
Exploring
animal behavior vector
Freely moving
shuffling k times
Other neurons
SINs
SANs
null distribution N
B
TNs
PNs
INs
Pre Post
SANs
SINs
Avg ΔF/F
Time (s)
C
Avg ΔF/F
Pre Post Pre Post
D
Signal-Behavior correlation (abs)
Correlation coefficient
TNs PNs INs
E
The engage estimation of SANs and SINs ensembles
Consistency (%)
Percentage of neurons (%)
TNs PNs INs

Fig. S3. Dynamics, correlation, and engagement analysis of functional ensembles.

(A) The diagram of subgrouping algorithm that divided neuron populations into SAN, SIN functional ensembles and other neurons.

(B) Event average ΔF/F calcium traces of SANs and SINs among the three types of neurons (TNs, PNs, and INs) at the end of social exploration (± 4 s). Social exploration ended at 0 seconds. The data are presented as the mean ± SEM (the line and shadow area). Red, SANs; blue, SINs. n = 11 mice (TNs), n = 6 mice (PNs), and n = 11 mice (INs), respectively.

(C) Comparison of the ΔF/F values before (-2.2 ~ -1.8 s) and after (+1.8 ~ +2.2 s) onset. The gray lines represent the relative ΔF/F change of individual mice, and the red and blue lines show the average changes of SANs and SINs. n = 11 mice (TNs), n = 6 mice (PNs), and n = 11 mice (INs), respectively. The Wilcoxon matched-pairs signed-rank test. *p < 0.05, **p < 0.01, ***p < 0.001; *ns*, no significance (p > 0.05). The data are shown as the mean ± SEM.

(D) Statistical analysis of the absolute Signal-Behavior correlation coefficient for all neurons in the FOV. n = 11 mice (TNs), n = 6 mice (PNs), and n = 11 mice (INs), respectively. One-way ANOVA followed by the Bonferroni *post hoc* test. The data are shown as the mean ± SEM. *p < 0.05, **p < 0.01, ***p < 0.001; *ns*, no significance (p > 0.05).

(E) Engagement analysis of social exploration events of SAN and SIN ensembles among the three types of neurons. In the box plots, the data are organized as the median and the 25th-75th percentile; the whiskers denote the maximal and the minimal values. Dunn's multiple comparisons test. **p < 0.01, ***p < 0.001; *ns*, no significance (p > 0.05). The values are shown as the mean ± SEM. For detailed statistical information, see Table S1.

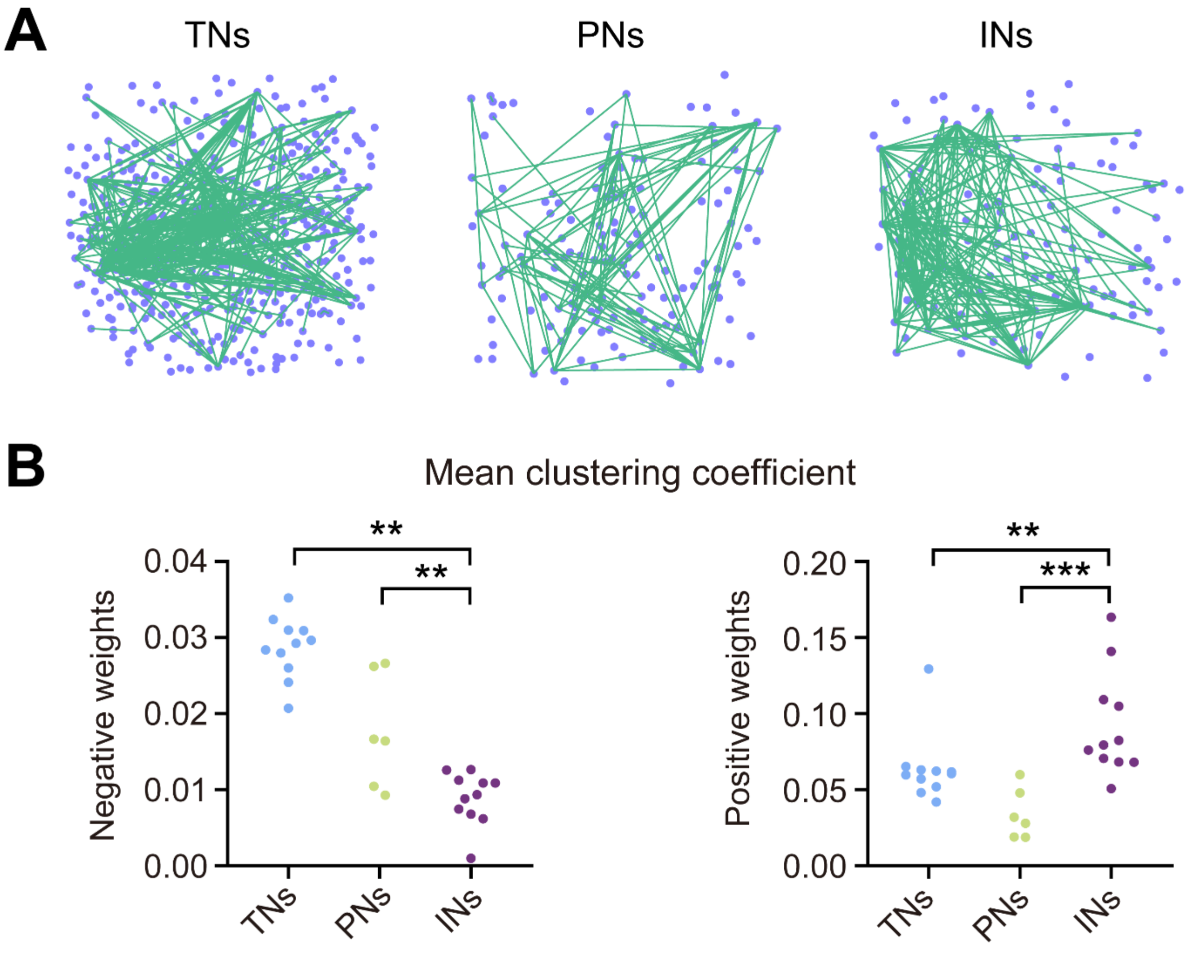

Fig. S4. Complex network properties of TNs, PNs, and INs.

(A) Typical neuronal functional connection graphs and the spatial distribution of neurons during social exploration. Each dot represents a neuron; each line indicates a connection between two neurons with a strength greater than 0.30. Left: TNs, middle: PNs, right: INs.

(B) Statistical analysis of the mean clustering coefficient in the neuronal functional connection graphs in WT mice during social exploration. Outliers were identified by the ROUT method and excluded from statistical analysis. One-way ANOVA followed by the Bonferroni *post hoc* test. The data are shown as the mean ± SEM. *$p < 0.05$, **$p < 0.01$, ***$p < 0.001$.

For detailed statistical information, see Table S1.

Fig. S5

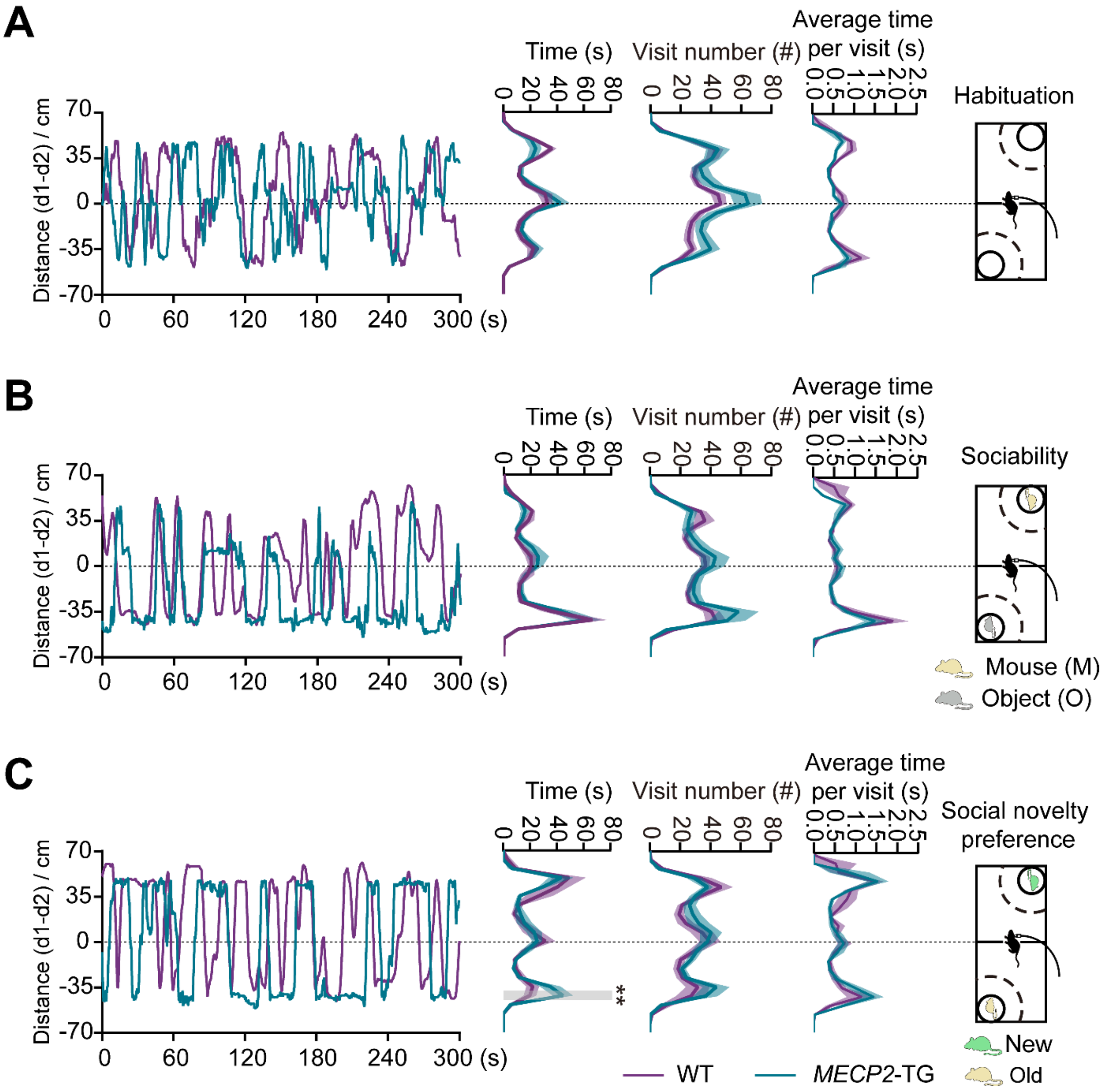

Fig. S5. Kymographs and analysis of mouse location in the two-chamber test.

(A to C) The kymograph displays the automatically tracked locations of the mice in 5 min habituation/sociability/social novelty preference trials. The time panel illustrates the time spent in the track zone. The visit number panel illustrates the shuttle number in the track zone. The average time per visit panel illustrates the time that the shuttling mice stayed in the track zone. Two-way RM ANOVA followed by the Bonferroni *post hoc* test. The data were shown as the mean ± SEM. **p < 0.01. Purple, WT mice (n = 11); dark green, *MECP2*-TG mice (n = 13).

Fig. S6

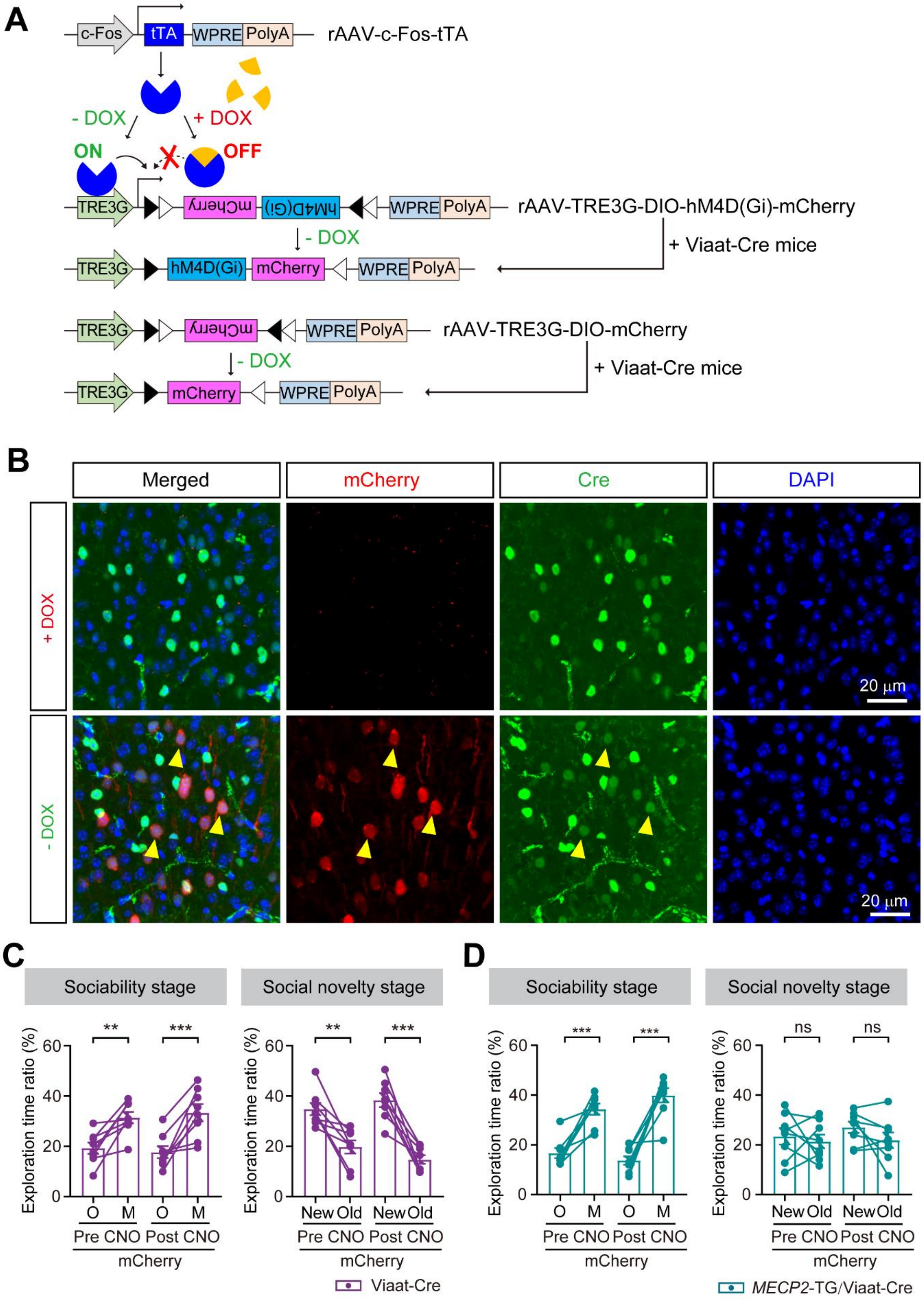
A
c-Fos
tTA
WPRE
PolyA
rAAV-c-Fos-tTA
- DOX
+ DOX
ON
OFF
TRE3G
mCherry
hM4D(Gi)
rAAV-TRE3G-DIO-hM4D(Gi)-mCherry
+ Viaat-Cre mice
rAAV-TRE3G-DIO-mCherry
B
Merged
mCherry
Cre
DAPI
+ DOX
- DOX
20 μm
C
Sociability stage
Social novelty stage
Exploration time ratio (%)
O
M
New
Old
Pre CNO
Post CNO
Viaat-Cre
D
ns
MECP2-TG/Viaat-Cre

Fig. S6. Schematic diagram and functional validation of Tet-Off and Cre-dependent chemogenetic viral system.

(A) Schematic diagram of the Tet-Off and DIO version of the chemogenetic viral system. The Viaat-Cre mice were injected with AAV9-c-Fos -tTA and AAV9-TRE-DIO-hM4D(Gi)-mCherry and AAV9-TRE-DIO-mCherry virus simultaneously in the PrL region.

(B) Representative images of brain slices from Viaat-Cre mice after DOX treatment (OFF) and withdrawal (ON) immunostained with anti-Cre antibody. The mice were injected with chemogenetic AAV virus 28 days earlier and *i.p.* injected with CNO for 0.5 h before histological assay. All social exploration activated neurons (mCherry+, red) are GABAergic neurons (Cre+, green) in DOX withdrawal (ON) mice (indicated with yellow arrowheads). No mCherry+ neurons were detected in the brain after DOX treatment (OFF).

(C and D) Exploration time ratio (%) of mCherry control viruses injected Viaat-Cre (C) and *MECP2*-TG/Viaat-Cre (D) mice in two-chamber sociability and social novelty preference assays, respectively. There was no any change before and after CNO administration.

Two-way RM ANOVA followed by the Bonferroni *post hoc* test. The data were shown as the mean ± SEM. $^{**}p < 0.01$, $^{***}p < 0.01$. Viaat-Cre mice ($n = 8$), and *MECP2*-TG/Viaat-Cre mice ($n = 8$).

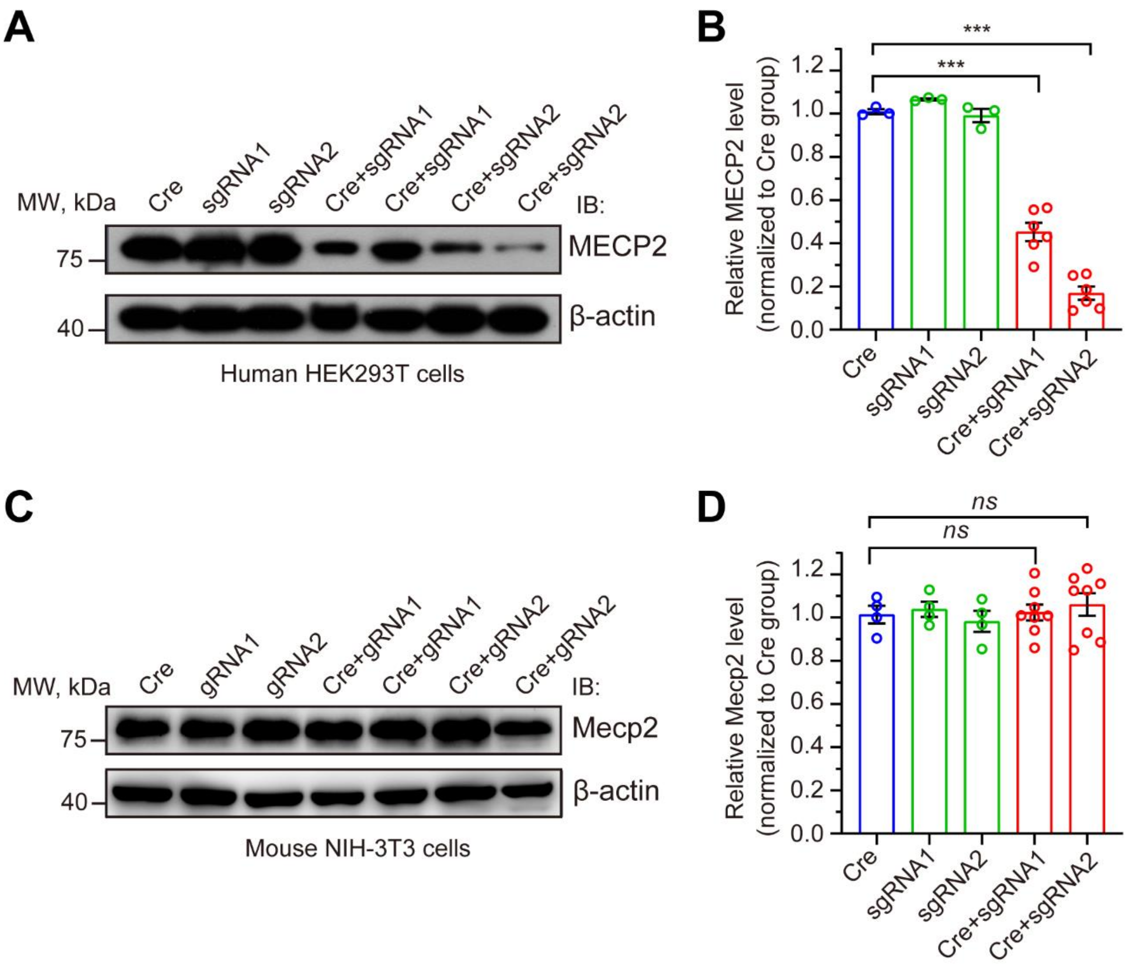

Fig. S7. Specific suppression of human *MECP2* but not mouse *Mecp2* gene by AAV-sgRNA *in vitro*.

(A) Western blot analysis of endogenous human MECP2 expression 48 h after AAV-sgRNA plasmids transfection in human-derived HEK293T cells.

(B) Quantitative analysis of human MECP2 expression in transfected HEK293T cells. Cells transfected with AAV-sgRNA1 and AAV-sgRNA2 plasmids showed significantly decreased human MECP2 expression compared to control cells. n = 6 in the AAV-sgRNA1 and AAV-sgRNA2 transfected cells, n = 3 in control cells. One-way ANOVA followed by the Bonferroni *post hoc* test. The data are shown as the mean ± SEM. ***$p < 0.001$.

(C) Western blot analysis of endogenous mouse Mecp2 expression 48 h after AAV-sgRNA plasmids transfection in mouse-derived NIH-3T3 cells.

(D) Quantitative analysis of endogenous mouse Mecp2 expression in transfected NIH-3T3 cells. Cells transfected with AAV-sgRNA1 or AAV-sgRNA2 plasmids showed no apparent differences

compared to control cells. n = 8 in the AAV-sgRNA1 and AAV-sgRNA2 transfected cells, n = 4 in control cells. One-way ANOVA followed by the Bonferroni *post hoc* test. The data are shown as the mean ± SEM. *ns*, no significance ($p > 0.05$).
For detailed statistical information, see Table S1.

Fig. S8

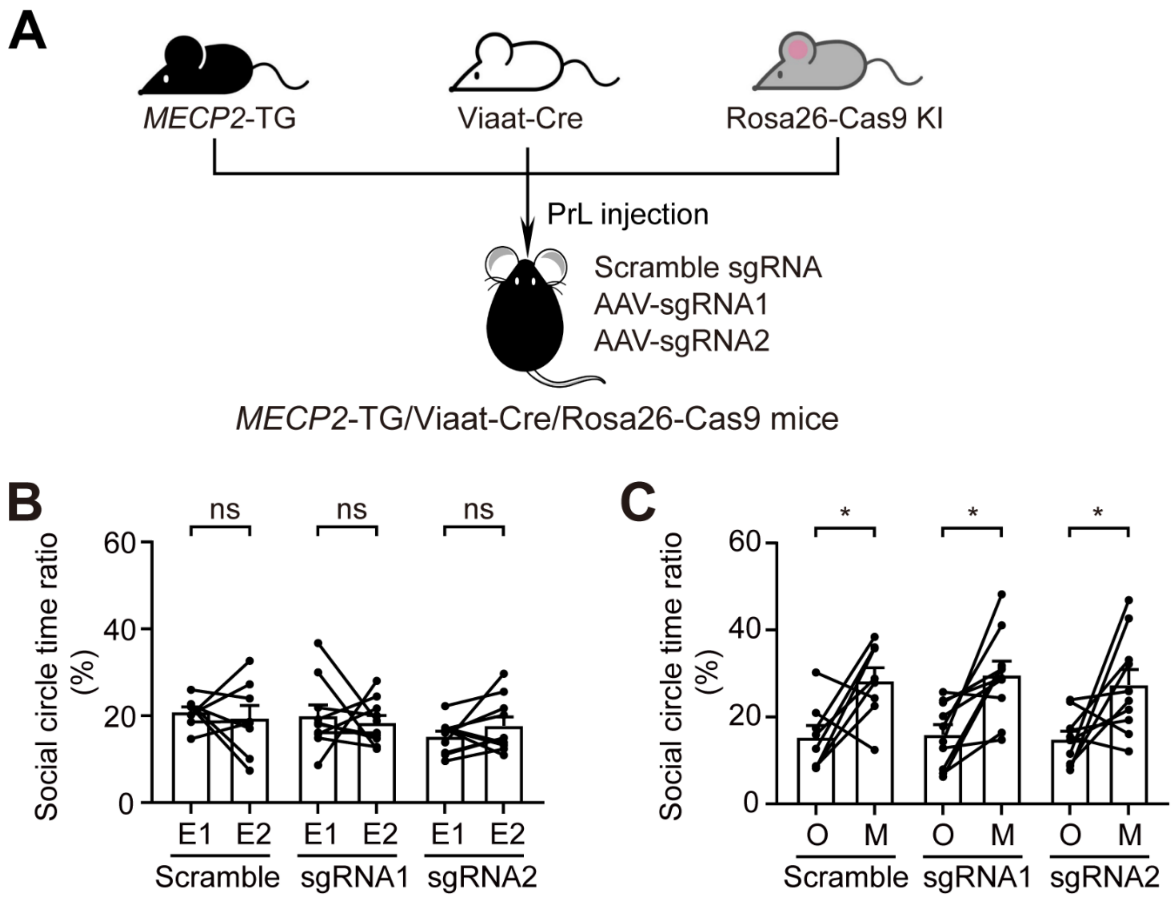

Fig. S8. The habituation and sociability performance in *MECP2*-TG mice was not affected by restoring MeCP2 levels within the PrL INs.

(A) Schematic diagram of generation of *MECP2*-TG/Viaat-Cre/Cas9-TG triple transgenic mice.

(B and C) Quantitative analysis shows no significant difference of the social circle time ratio during habituation and sociability performance in AAV-sgRNA1, AAV-sgRNA2 and control scramble sgRNA infected triple transgenic mice.

The data are shown as the mean ± SEM. Two-way RM ANOVA followed by the Bonferroni *post hoc* test. *$p < 0.05$, *ns*, no significance ($p > 0.05$).

For detailed statistical information, see Table S1.

Fig. S9

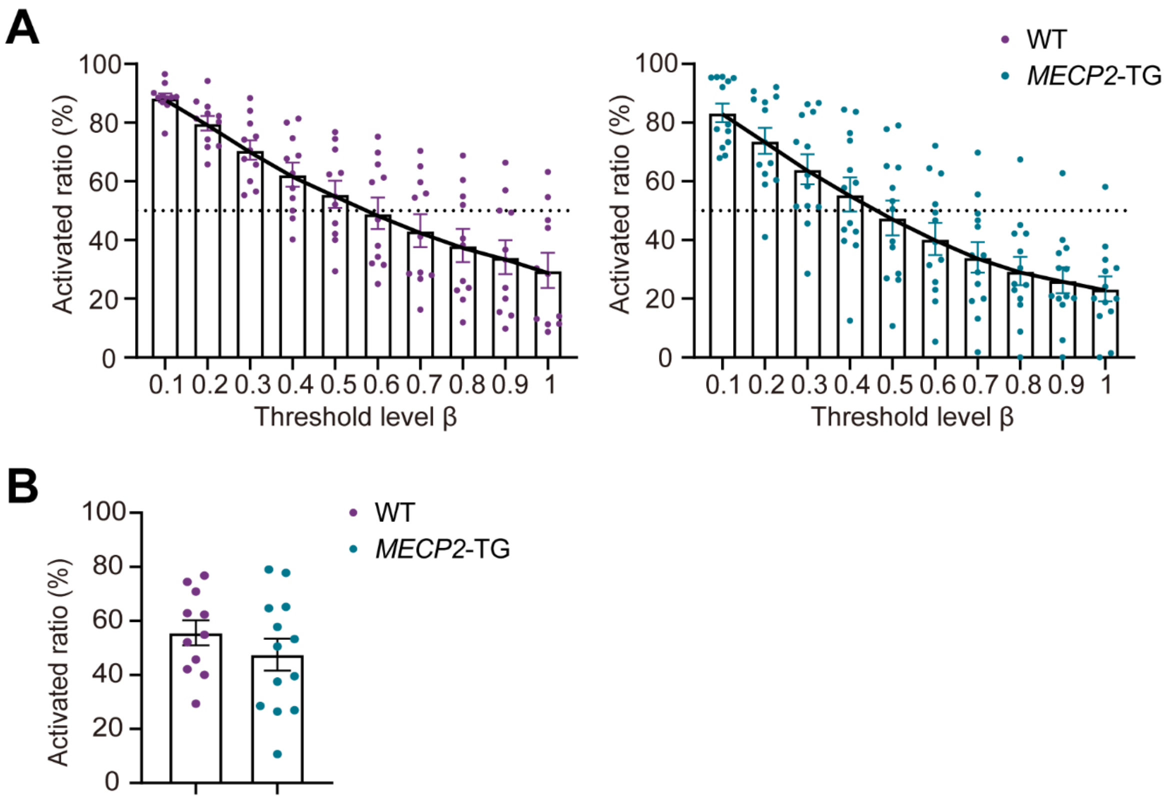

Fig. S9. The effect of thresholding and the resulting activated ratios of EANs.

(A) The effect of β selection on the activated ratio. A neuron was considered an EAN if it exhibited an average ΔF/F value in one specific exploration (E) state β times higher than the noise level of the NE state. The activated ratio was defined as the ratio between the number of EANs and all neurons in an individual mouse. Dotted line, activated ratio = 50%. Purple, WT mice (n = 11); dark green, *MECP2*-TG mice (n = 13).

(B) Statistical analysis of the activated ratio in WT and *MECP2*-TG mice at β = 0.5. Unpaired *t* test. No significant difference was found.

For detailed statistical information, see Table S1.

Fig. S10

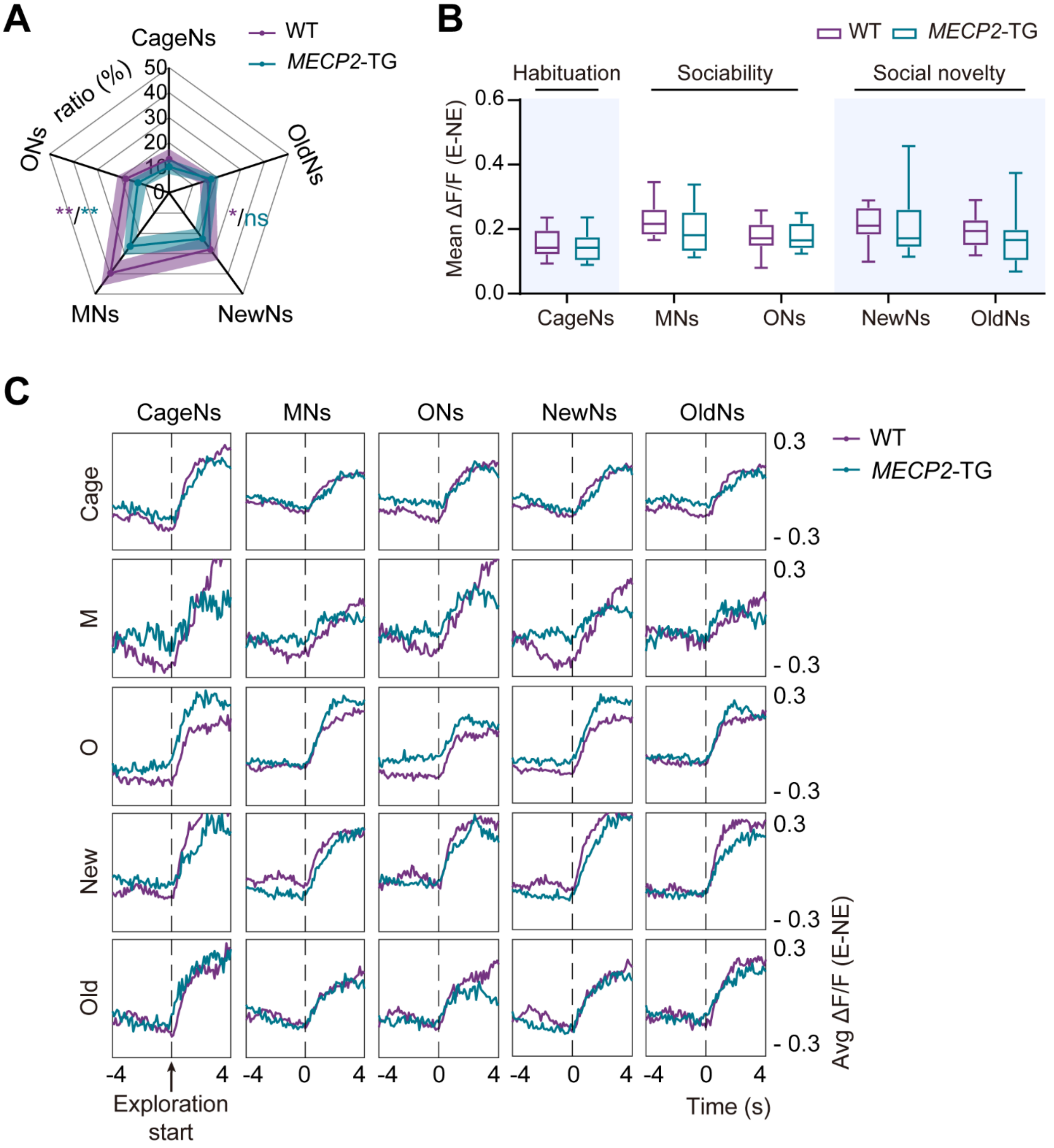

Fig. S10. Ratio, mean relative ΔF/F and dynamic features of EANs.

(A) Radar plot of the ratio of each kind of EAN relative to total EANs in the five exploration states. Paired t test. The data are shown as the mean ± SEM.

(B) Box plot of the mean relative ΔF/F values of the five kinds of EANs in their corresponding exploration states. In the box plots, the data are organized as the median and the 25th-75th percentile;

the whiskers denote the maximal and the minimal values. Unpaired *t* test. The values are shown as the mean ± SEM.

(C) The event average relative ΔF/F calcium traces of EANs at exploration onset. Each row represents an exploration state. The dotted line indicates the onset of exploration. Purple, WT mice (n = 11); dark green, *MECP2*-TG mice (n = 13).

For detailed statistical information, see Table S1.

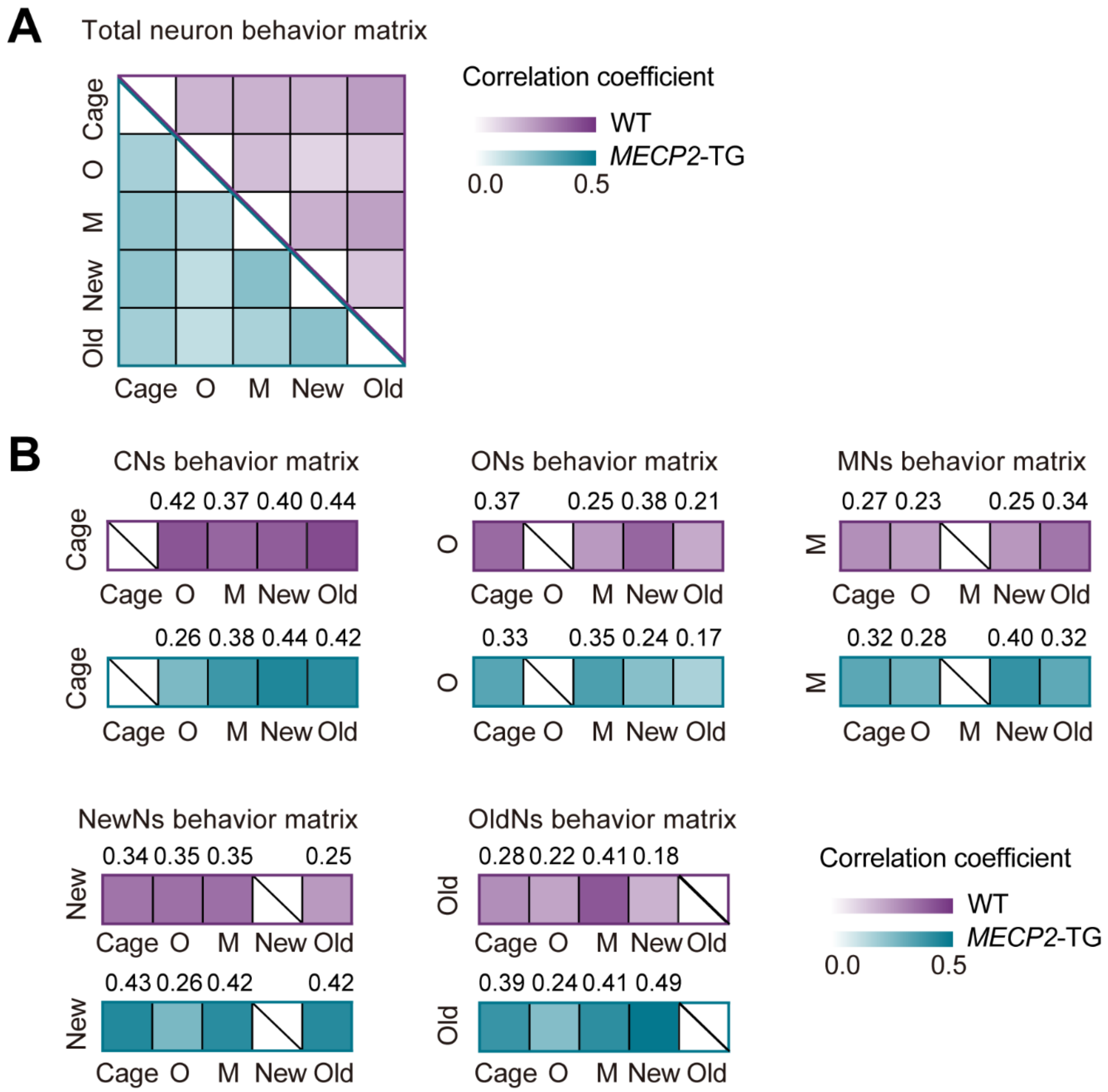

Fig. S11. Average behavioral correlation matrix of EANs and total neurons.

(A) Average behavioral correlation matrix of the calcium activity of total neurons during different exploration behaviors. The correlation values of all blocks were less than 0.3. Purple, WT mice (n = 11); dark green, *MECP2*-TG mice (n = 13).

(B) Average behavioral correlation matrix of the calcium activity of CageNs, MNs, ONs, NewNs, and OldNs during different exploration behaviors. Purple, WT mice (n = 11); dark green, *MECP2*-TG mice (n = 13).

Fig. S12

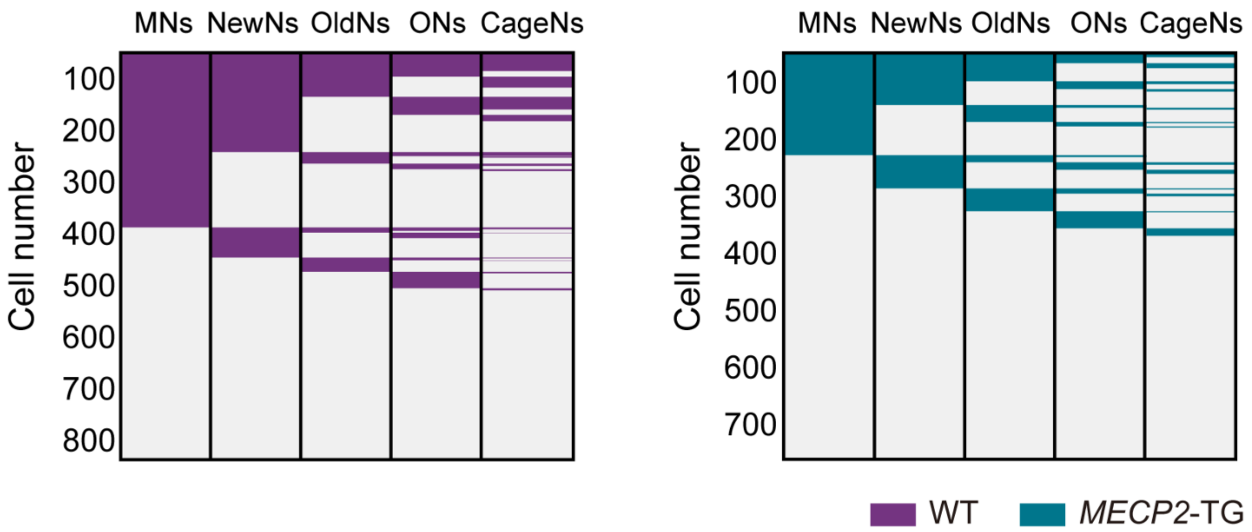

Fig. S12. Neuronal labeling in WT and *MECP2*-TG mice.

Schematic 'barcode' of the activation patterns of neurons. Each neuron was labeled according to its response to five direct exploration behaviors. Purple, neurons in WT mice (n = 817); dark green, neurons in *MECP2*-TG mice (n = 788).

Fig. S13

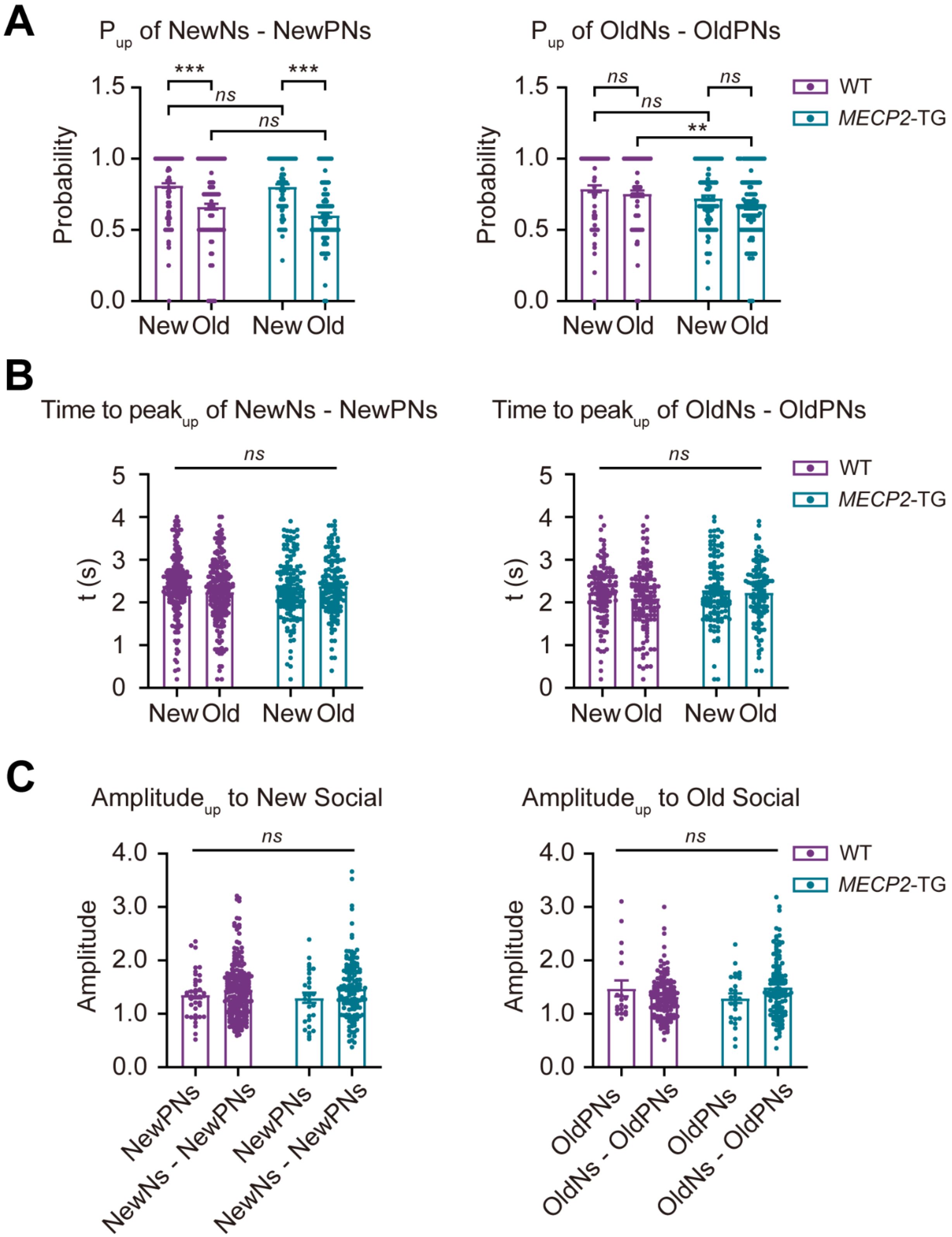
A
P up of NewNs - NewPNs
P up of OldNs - OldPNs
Probability
1.5
1.0
0.5
0.0
***
ns
**
New Old New Old
WT
MECP2-TG
B
Time to peak up of NewNs - NewPNs
Time to peak up of OldNs - OldPNs
t (s)
5
4
3
2
1
0
C
Amplitude up to New Social
Amplitude up to Old Social
Amplitude
4.0
3.0
2.0
1.0
0.0
NewPNs
NewNs - NewPNs
OldPNs
OldNs - OldPNs

Fig. S13. The Ca^{2+} dynamics of EANs in socially activated epochs.

(A) The activation probability of NewNs (except NewPNs) and OldNs (except OldPNs) in all new mouse exploration-activated epochs (new) and old mouse exploration-activated epochs (old). NewNs (except NewPNs) always had a higher probability of activation during new mouse exploration than during old mouse exploration in both WT and *MECP2*-TG mice. OldNs (except OldPNs) were activated similarly with comparable probability in response to new and old social cues. Two-way RM ANOVA followed by the Bonferroni *post hoc* test. The data are shown as the mean ± SEM. *p < 0.05, **p < 0.01, ***p < 0.001; *ns*, no significance (p > 0.05).

(B) The rise time of NewNs (except NewPNs) and OldNs (except OldPNs) in socially activated epochs. No difference was found among any of the groups. Two-way RM ANOVA followed by the Bonferroni *post hoc* test. The data is shown as the mean ± SEM. *ns*, no significance (p > 0.05).

(C) Comparison of the extent of the increase of some social cue-associated neurons and social cue-preferred interneurons. No difference was found among any of the groups. Two-way RM ANOVA followed by the Bonferroni *post hoc* test. The data are shown as the mean ± SEM. *ns*, no significance (p > 0.05).

For detailed statistical information, see Table S1.

Fig. S14

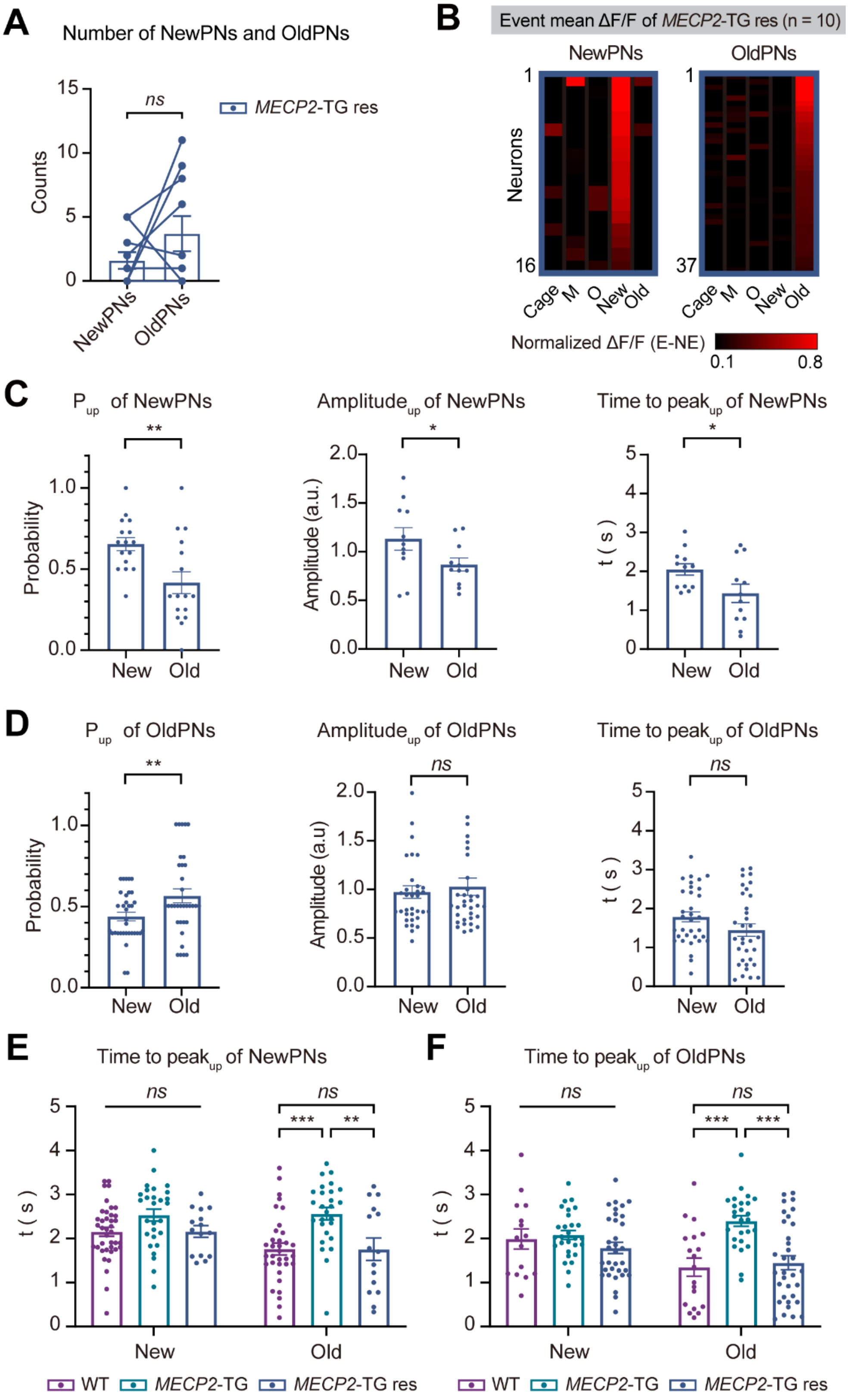

A
Number of NewPNs and OldPNs
ns
MECP2-TG res
Counts
NewPNs
OldPNs
B
Event mean ΔF/F of MECP2-TG res (n = 10)
NewPNs
OldPNs
Neurons
Cage M O New Old
Normalized ΔF/F (E-NE)
0.1
0.8
C
P up of NewPNs
Amplitude up of NewPNs
Time to peak up of NewPNs
Probability
Amplitude (a.u.)
t (s)
New Old
D
P up of OldPNs
Amplitude up of OldPNs
Time to peak up of OldPNs
Amplitude (a.u)
E
Time to peak up of NewPNs
F
Time to peak up of OldPNs
New
Old
WT
MECP2-TG
MECP2-TG res

Fig. S14. The Ca^{2+} dynamics of NewPNs and OldPNs in socially activated epochs in *MECP2*-TG rescue mice.

(A) The number of NewPNs and OldPNs in *MECP2*-TG rescue mice (*MECP2*-TG res, indicated by deep blue dots) (n = 10). Paired *t*-test. *ns*, no significance.

(B) Heatmaps of the event mean ΔF/F values of NewPNs and OldPNs in five different exploration states. Intensity was normalized by the maximum value across all exploration states. Each row represents a NewPN or an OldPN, and each column represents an exploration state. Left panel: NewPNs (n = 16); Right panel: OldPNs (n = 37).

(C and D) The parametric estimation of social cue activated epochs of NewPNs and OldPNs. All amplitude values were normalized by the mean value under social cue activated epochs. Paired *t*-test. Any social cue-preferred interneurons that did not show an activated epoch under its corresponding social cue were eliminated. *$p < 0.05$, **$p < 0.01$, ***$p < 0.001$; *ns*, no significance.

(E and F) The comparison of rise time of NewPNs and OldPNs between WT, *MECP2*-TG and *MECP2*-TG res mice. Purple dots, WT mice (n = 11); dark green dots, *MECP2*-TG mice (n = 13); deep blue dots, *MECP2*-TG-res mice (n = 10). One-way ANOVA followed by the Bonferroni *post hoc* test. For detailed statistical information, see Table S1.

Suppl. Table S1: Summary of detailed statistical analysis

Fig.	Sample (mean ± SEM)	Sample number	Statistical methods	P value
**Fig. 1J**	For SANs, TNs_{pre} (0.32 ± 0.02) vs. TNs_{post} (0.34 ± 0.02)	$n_{(TNs)}$ = 11 mice	Paired Student's t-test	t=5.270, df=10, p = 0.0004
	For SANs, PNs_{pre} (0.16 ± 0.02) vs. PNs_{post} (0.30 ± 0.07)	$n_{(PNs)}$ = 6 mice	Wilcoxon matched-pairs signed-rank test	W = 21.00, p = 0.0313
	For SANs, INs_{pre} (0.46 ± 0.04) vs. INs_{post} (0.57 ± 0.04)	$n_{(INs)}$ = 11 mice	Paired Student's t-test	t=3.319, df=10, p = 0.0078
	For SINs, TNs_{pre} (0.26 ± 0.01) vs. TNs_{post} (0.25 ± 0.04)	$n_{(TNs)}$ = 11 mice	Paired Student's t-test	t=7.697, df=10, p < 0.0001
	For SINs, PNs_{pre} (0.25 ± 0.10) vs. PNs_{post} (0.18 ± 0.03)	$n_{(PNs)}$ = 6 mice	Wilcoxon matched-pairs signed-rank test	W = -21.00, p = 0.0313
	For SINs, INs_{pre} (0.47 ± 0.03) vs. INs_{post} (0.40 ± 0.03)	$n_{(INs)}$ = 11 mice	Paired Student's t-test	t=5.995, df=10, p = 0.0001
**Fig. 1K**	For SAN, INs (62.30 ± 4.90 %) vs. TNs (27.52 ± 2.06 %);<br>INs (62.30 ± 4.90 %) vs. PNs (31.56 ± 1.97 %)	$n_{(TNs)}$ = 11 mice,<br>$n_{(INs)}$ = 11 mice,<br>$n_{(PNs)}$ = 6 mice	one-way ANOVA with Bonferroni post hoc test	F (2, 25) = 29.04,<br>P < 0.0001.<br>Post hoc test:<br>p < 0.0001,<br>p < 0.0001
	For SIN, INs (20.72 ± 3.11 %) vs. TNs (40.27 ± 2.61 %);<br>INs (20.72 ± 3.11 %) vs. PNs (34.31 ± 5.95%)	$n_{(TNs)}$ = 11 mice,<br>$n_{(INs)}$ = 11 mice,<br>$n_{(PNs)}$ = 6 mice	one-way ANOVA with Bonferroni post hoc test	F (2, 25) = 9.429,<br>P = 0.0009.<br>Post hoc test:<br>p = 0.0005,<br>p = 0.0390

**Fig. 1L**	For SANs, INs (0.13 ± 0.01) *vs.* TNs (0.06 ± 0.00); INs (0.13 ± 0.01) *vs.* PNs (0.09 ± 0.01); TNs (0.06 ± 0.00) *vs.* PNs (0.09 ± 0.01)	n $_{(TNs)}$ = 11 mice, n $_{(INs)}$ = 11 mice, n $_{(PNs)}$ = 6 mice	one way ANOVA with Bonferroni post hoc test	F (2, 25) = 32.45, p < 0.0001. Post hoc test: p < 0.0001, p = 0.0012, p = 0.0420
	For SINs, INs (-0.08 ± 0.00) *vs.* TNs (-0.06 ± 0.00); INs (-0.08 ± 0.00) *vs.* PNs (-0.06 ± 0.00); TNs (-0.06 ± 0.00) *vs.* PNs (-0.06 ± 0.00)	n $_{(TNs)}$ = 11 mice, n $_{(INs)}$ = 11 mice, n $_{(PNs)}$ = 6 mice	one way ANOVA with Bonferroni post hoc test	F (2, 25) = 16.16, p < 0.0001. Post hoc test: p < 0.0001, p = 0.0007, p > 0.9999
**Fig. 2C**	M (31.93 ± 3.17%) *vs.* O (6.86 ± 1.32%) in WT mice; M (31.57 ± 5.09%) *vs.* O (5.69 ± 0.84%) in *MECP2*-TG mice	n $_{(WT)}$ = 11 mice; n $_{(MECP2\text{-}TG)}$ = 13 mice	Two-way RM ANOVA with Bonferroni post hoc test	Factor 1 (genotype): F (1, 22) = 0.05279, P = 0.8204; Factor 2 (object): F (1, 22) = 66.58, P<0.0001; Factor interaction: F (1, 22) = 0.01671, P=0.8983. Post hoc test: p < 0.0001, p < 0.0001
**Fig. 2D**	Old (9.92 ± 1.47%) *vs.* New (25.21 ± 3.69%) in WT mice Old (20.88 ± 3.14%) *vs.* New (19.70 ± 2.50%) in *MECP2*-TG mice	n $_{(WT)}$ = 11 mice; n $_{(MECP2\text{-}TG)}$ = 13 mice	Two-way RM ANOVA with Bonferroni post hoc test	Factor 1 (genotype): F (1, 22) = 0.6749, P = 0.4202; Factor 2 (object): F (1, 22) = 9.629, P<0.0052; Factor interaction: F

	WT Old (9.92 ± 1.47%) *vs.* *MECP2*-TG Old (20.88 ± 3.14%)			(1, 22) = 13.11, P=0.0015. Post hoc test: p = 0.0003, p > 0.9999, p = 0.0185
**Fig. 2G**	Before CNO administration, M (39.60 ± 3.44%) *vs.* O (12.95 ± 1.33%) in WT mice After CNO administration, M (31.92 ± 4.15%) *vs.* O (19.56 ± 4.50%) in WT mice	n $_{(WT)}$ = 10 mice	Two-way RM ANOVA with Bonferroni post hoc test	Factor 1 (drug): F (1, 18) = 0.0663, P = 0.7997; Factor 2 (object): F (1, 18) = 17.98, P = 0.0005; Factor interaction: F (1, 18) = 2.412, P = 0.1378. Post hoc test: p = 0.0014, p = 0.1471
**Fig. 2H**	Before CNO administration, M (42.13 ± 5.29%) *vs.* O (9.39 ± 1.25%) in *MECP2*-TG mice After CNO administration, M (35.70 ± 5.28%) *vs.* O (23.34 ± 6.27%) in *MECP2*-TG mice	n $_{(MECP2\text{-}TG)}$ = 7 mice	Two-way RM ANOVA with Bonferroni post hoc test	Factor 1 (drug): F (1, 12) = 1.224, P = 0.2902; Factor 2 (object): F (1, 12) = 13.81, P = 0.0029; Factor interaction: F (1, 12) = 2.817, P = 0.1191. Post hoc test: p = 0.0049, p = 0.3504
**Fig. 2I**	Before CNO administration, Old (16.76 ± 1.82%) *vs.* New (34.43 ± 3.16%) in WT mice After CNO administration, Old (21.94 ± 3.16%) *vs.* New (25.77 ± 3.59%) in WT mice	n $_{(WT)}$ = 10 mice	Two-way RM ANOVA with Bonferroni post hoc test	Factor 1 (drug): F (1, 18) = 0.4733, P = 0.5003; Factor 2 (object): F (1, 18) = 9.861, P = 0.0057; Factor interaction: F (1, 18) = 4.084, P = 0.0584. Post hoc test:

				p = 0.0037,<br>p = 0.8780
**Fig. 2J**	Before CNO administration, Old (20.99 ± 2.35%) *vs.* New (25.70 ± 2.94%) in *MECP2*-TG mice<br>After CNO administration, Old (22.20 ± 4.02%) *vs.* New (34.58 ± 6.71%) in *MECP2*-TG mice	n $_{(MECP2\text{-TG})}$ = 7 mice	Two-way RM ANOVA with Bonferroni post hoc test	Factor 1 (drug): F (1, 12) = 3.084, P = 0.1045; Factor 2 (object): F (1, 12) = 2.479, P = 0.1413; Factor interaction: F (1, 12) = 0.5004, P = 0.4929.<br>Post hoc test:<br>p > 0.9999,<br>p = 0.2652
**Fig. 3E**	For scramble group, New mouse (New) (21.23 ± 1.93) *vs.* Old mouse (Old) (22.12 ± 3.58);<br>For sgRNA1 group, New (31.93 ± 3.52) *vs.* Old (14.66 ± 2.65);<br>For sgRNA2 group, New (26.80 ± 2.85) *vs.* Old (12.70 ± 2.18)	n $_{(Scramble)}$ = 8 mice,<br>n $_{(sg1)}$ = 10 mice,<br>n $_{(sg2)}$ = 10 mice	Two-way RM ANOVA with Bonferroni post hoc test.	Factor 1 (sgRNA): F (2, 25) = 1.685, P = 0.2058; Factor 2 (object): F (1, 25) = 12.37, P = 0.0017; Factor interaction: F (2, 25) = 3.504, P = 0.0456.<br>Post hoc test:<br>p > 0.9999,<br>p = 0.0042,<br>p = 0.0213
**Fig. 4C**	For WT group, NewPN (3.55 ± 1.25) *vs.* OldPN (2.00 ± 0.95);<br>For *MECP2*-TG group, NewPN (2.23 ± 0.98) *vs.* OldPN (2.23 ± 0.68).	n $_{(WT)}$ = 11 mice;<br>n $_{(MECP2\text{-TG})}$ = 13 mice	Two-way RM ANOVA followed by Bonferroni post hoc test	Factor 1 (genotype): F (1, 22) = 0.2906, P = 0.5952; Factor 2 (neuron): F (1, 22) = 0.6915, P = 0.4146; Factor interaction: F (1, 22) =

				0.6915, P = 0.4146. Post hoc test: p = 0.5414, p > 0.9999
**Fig. 4H**	For NewPN, WT New (0.69 ± 0.04) *vs.* WT Old (0.44± 0.03); *MECP2*-TG New (0.75 ± 0.03) *vs.* *MECP2*-TG Old (0.51 ± 0.05)	n (NewPN of WT) = 39; n (NewPN of *MECP2*-TG) = 28	Two-way RM ANOVA with Bonferroni post hoc test	Factor 1 (genotype): F (1, 65) = 3.5000, P = 0.0659; Factor 2 (object): F (1, 65) = 40.49, P < 0.0001; Factor interaction: F (1, 65) = 0.0363, P = 0.8495. Post hoc test: p < 0.001, p = 0.0003
	For OldPN, WT New (0.47 ± 0.08) *vs.* WT Old (0.64 ± 0.05); *MECP2*-TG New (0.54 ± 0.05) *vs.* *MECP2*-TG Old (0.67 ± 0.03)	n (OldPN of WT) = 19; n (OldPN of *MECP2*-TG) = 26	Two-way RM ANOVA with Bonferroni post hoc test	Factor 1 (genotype): F (1, 43) = 0.6889, P = 0.4111; Factor 2 (object): F (1, 43) = 12.11, P =0.0012; Factor interaction: F (1, 43) = 0.1295, P = 0.7207. Post hoc test: p = 0.0306, p = 0.0415
**Fig. 4I**	For NewPN, WT New (1.10 ± 0.05) *vs.* WT Old (0.97 ± 0.05); *MECP2*-TG New (1.11 ± 0.08) *vs.* *MECP2*-TG Old (0.78 ± 0.08)	n (NewPN of WT to New) = 35; n (NewPN of WT to Old) = 33; n (NewPN of *MECP2*-TG to New) = 27, n (NewPN of *MECP2*-TG to Old) = 26	Two-way ANOVA with Bonferroni post hoc test	Factor 1 (genotype): F (1, 61) = 0.8520, P = 0.3596; Factor 2 (object): F (1, 56) = 28.4700, P < 0.0001; Factor interaction: F

				(1, 56) = 4.5410, P = 0.0375. Post hoc test: p = 0.0365, p < 0.0001
	For OldPN, WT New (0.91 ± 0.08) *vs.* WT Old (1.15 ± 0.13); *MECP2*-TG New (1.02 ± 0.09) *vs.* *MECP2*-TG Old (1.06 ± 0.08)	n (OldPN of WT to New) = 14, n (OldPN of WT to Old) = 18; n (OldPN of *MECP2*-TG to New) = 27, n (OldPN of *MECP2*-TG to Old) = 25	Two-way ANOVA with Bonferroni post hoc test	Factor 1 (genotype): F (1, 45) = 0.0044, P = 0.9475; Factor 2 (object): F (1, 35) = 6.4640, P = 0.0156; Factor interaction: F (1, 35) = 1.8860, P = 0.1784. Post hoc test: p = 0.0399, p = 0.6673
**Fig. 4J**	For NewPN, WT New (2.16 ± 0.10) *vs.* WT Old (1.76 ± 0.13); *MECP2*-TG New (2.53 ± 0.14) *vs.* *MECP2*-TG Old (2.57 ± 0.14); WT Old (1.76 ± 0.13) *vs.* *MECP2*-TG Old (2.57 ± 0.14)	n (NewPN of WT to New) = 39; n (NewPN of WT to Old) = 34; n (NewPN of *MECP2*-TG to New) = 28, n (NewPN of *MECP2*-TG to Old) = 27	Two-way ANOVA with Bonferroni post hoc test.	Factor 1 (genotype): F (1, 66) = 20.00, P < 0.0001; Factor 2 (object): F (1, 58) = 2.151, P = 0.1479; Factor interaction: F (1, 58) = 2.864, P = 0.0960. Post hoc test: p = 0.0387, p > 0.9999, p < 0.0001
	For OldPN, WT New (1.99 ± 0.23) *vs.* WT Old (1.35 ± 0.21); *MECP2*-TG New (2.08 ± 0.10) *vs.*	n (OldPN of WT to New) = 15, n (OldPN of WT to Old) = 19; n (OldPN of *MECP2*-TG to New) = 28, n	Two-way ANOVA with Bonferroni post hoc test.	Factor 1 (genotype): F (1, 47) = 11.75, P = 0.0013; Factor 2 (object): F (1, 37) = 1.421, P = 0.2409;

	MECP2-TG Old (2.40 ± 0.12); WT Old (1.35 ± 0.21) vs. *MECP2*-TG Old (2.40 ± 0.12)	(OldPN of *MECP2*-TG to Old) = 26		Factor interaction: F (1, 37) = 11.68, P = 0.0016. Post hoc test: p = 0.0117, p = 0.1575, p < 0.0001
**Supplementary Fig. S1A**	With 5g weight (11.62 ± 1.23, n = 8) *vs.* W/O 5g weight (12.76 ± 1.30, n = 8)	n = 8<br>n = 8	Two-tailed paired t-test	t = 0.6410, df = 14<br>p = 0.5319
**Supplementary Fig. S2A**	For TNs, M (28.17 ± 2.13%) *vs.* O (10.71 ± 1.03%);<br>For PNs, M (28.12 ± 3.35%) *vs.* O (10.76 ± 2.53%);<br>For INs, M (31.93 ± 3.17%) *vs.* O (6.86 ± 1.32%).	n $_{(TNs)}$ = 11 mice, n $_{(PNs)}$ = 6 mice, n $_{(INs)}$ = 11 mice.	Two-way RM ANOVA with post hoc Bonferroni correction.	Factor 1 (genotype): F (2, 25) = 0.0002, P = 0.9998; Factor 2 (object): F (1, 25) = 102.4, P < 0.0001; Factor interaction: F (2, 25) = 1.943, P = 0.1643. Post hoc test: p <0.0001, p = 0.0008, p <0.0001
**Supplementary Fig. S2B**	TNs (0.72 ± 0.03) *vs.* PNs (0.73 ± 0.05); TNs (0.72 ± 0.03) *vs.* INs (0.82 ± 0.03); PNs (0.73 ± 0.05) *vs.* INs (0.82 ± 0.03)	n $_{(TNs)}$ = 11 mice, n $_{(PNs)}$ = 6 mice, n $_{(INs)}$ = 11 mice.	One-way ANOVA followed by Bonferroni post hoc test	F (2, 25) = 3.358, P = 0.0511. Post hoc test: p > 0.9999, p = 0.0649, p = 0.2537
**Supplementary Fig. S3B**	For SANs, TNs_{pre} (0.34 ± 0.02) *vs.* TNs_{post} (0.32 ± 0.02)	n $_{(TNs)}$ = 11 mice	Paired Student's t-test	t = 7.355, df = 10,<br>p < 0.0001
	For SANs, PNs_{pre} (0.34 ± 0.09) *vs.* PNs_{post} (0.23 ± 0.03);	n $_{(PNs)}$ = 6 mice	Wilcoxon matched-pairs signed-rank test	W = -19.00,<br>p = 0.0625

	For SANs, INs_{pre} (0.61 ± 0.04) *vs.* INs_{post} (0.42 ± 0.03)	n $_{(INs)}$ = 11 mice	Paired Student's t-test	t = 7.556, df = 10,<br>p < 0.0001
	For SINs, TNs_{pre} (0.23 ± 0.01) *vs.* TNs_{post} (0.27 ± 0.01);	n $_{(TNs)}$ = 11 mice	Paired Student's t-test	t = 6.511, df = 10,<br>p < 0.0001
	For SINs, PNs_{pre} (0.16 ± 0.02) *vs.* PNs_{post} (0.22 ± 0.03);	n $_{(PNs)}$ = 6 mice	Paired Student's t-test	t = 4.349, df = 5,<br>p = 0.0074
	For SINs, INs_{pre} (0.37 ± 0.03) *vs.* INs_{post} (0.43 ± 0.03)	n $_{(INs)}$ = 11 mice	Paired Student's t-test	T = 3.725, df=10,<br>p = 0.0039
**Supplementary Fig. S3C**	INs (0.10 ± 0.01) *vs.* TNs (0.05 ± 0.00);<br>INs (0.10 ± 0.01) *vs.* PNs (0.05 ± 0.01)	n $_{(TNs)}$ = 11 mice, n $_{(PNs)}$ = 6 mice, n $_{(INs)}$ = 11 mice.	one-way ANOVA followed by Bonferroni post hoc test.	F (2, 25) = 27.23, P < 0.0001.<br>Post hoc test:<br>p < 0.0001,<br>p < 0.0001
**Supplementary Fig. S3D**	Consistency of SAN:<br>TNs (32.82 ± 0.28%) *vs.* PNs (33.83 ± 0.88%);<br>TNs (32.82 ± 0.28%) *vs.* INs (66.46 ± 0.71%);<br>PNs (33.83 ± 0.88%) *vs.* INs (66.46 ± 0.71%);	n $_{(TNs)}$ = 579 neurons,<br>n $_{(PNs)}$ = 112 neurons,<br>n $_{(INs)}$ = 279 neurons;	Kruskal-Wallis test with Dunn's multiple comparisons test	Kruskal-Wallis statistic value = 591.6, p < 0.0001.<br>Dunn's multiple comparisons test:<br>p > 0.9999,<br>p < 0.0001,<br>p < 0.0001
	Consistency of SIN:<br>TNs (48.50 ± 0.35%) *vs.* PNs (54.82 ± 0.95%);<br>TNs (48.50 ± 0.35%) *vs.* INs (42.18 ± 1.09%);<br>PNs (54.82 ± 0.95%) *vs.* INs (42.18 ± 1.09%)	n $_{(TNs)}$ = 891 neurons,<br>n $_{(PNs)}$ = 108 neurons,<br>n $_{(INs)}$ = 90 neurons.	Kruskal-Wallis test with Dunn's multiple comparisons test	Kruskal-Wallis statistic value = 57.87, p < 0.0001.<br>Dunn's multiple comparisons test:<br>p < 0.0001,<br>p < 0.0001,<br>p < 0.0001
**Supplementary Fig. S3D**	Percentage of neurons for	n$_{(TNs)}$ = 56 events,	Kruskal-Wallis test with	Kruskal-Wallis statistic value =

	SAN: TNs (30.14 ± 1.07%) *vs.* PNs (34.83 ± 2.39%); TNs (30.14 ± 1.07%) *vs.* INs (62.03 ± 3.14%); PNs (34.83 ± 2.39%) *vs.* INs (62.03 ± 3.14%);	$n_{(PNs)}$ = 34 events, $n_{(INs)}$ = 41 events;	Dunn's multiple comparisons test	56.10, p < 0.0001. Dunn's multiple comparisons test: p = 0.6121, p < 0.0001, p < 0.0001
	Percentage of neurons for SIN: TNs (51.52 ± 1.28%) *vs.* PNs (58.80 ± 2.22%); TNs (51.52 ± 1.28%) *vs.* INs (39.29 ± 2.45%); PNs (58.80 ± 2.22%) *vs.* INs (39.29 ± 2.45%);	$n_{(TNs)}$ = 56 events, $n_{(PNs)}$ = 33 events, $n_{(INs)}$ = 41 events.	Kruskal-Wallis test with Dunn's multiple comparisons test	Kruskal-Wallis statistic value = 28.17, p < 0.0001. Dunn's multiple comparisons test: p = 0.0855, p = 0.0011, p < 0.0001
**Supplementary Fig. S4B**	For Negative weights, INs (0.01 ± 0.00) *vs.* TNs (0.03 ± 0.00); INs (0.01 ± 0.00) *v.s.* PNs (0.02 ± 0.00);	n_{mice}(TNs) = 11, n_{mice}(PNs) = 6, n_{mice}(INs) = 11,	Outliers were identified by ROUT method and excluded in statistic analyze. One-way ANOVA with Bonferroni post hoc test,	F (2, 25) = 48.70, P < 0.0001. Post hoc test: p < 0.0001, p = 0.0037
	For Positive weights, INs (0.09 ± 0.01) *vs.* TNs (0.06 ± 0.01); INs (0.09 ± 0. 01) *vs.* PNs (0.03 ± 0.01)	n_{mice}(TNs) = 11, n_{mice}(PNs) = 6, n_{mice}(INs) = 11,		F (2, 24) =12.66, P = 0.0002. Post hoc test: p = 0.0077, p = 0.0002
**Supplementary Fig. S6C, left panel**	Before CNO administration, M (31.46 ± 2.29%) *vs.* O (19.32 ± 2.24%) in WT mice After CNO administration, M (33.39 ± 3.43%) *vs.* O	$n_{(WT)}$ = 8 mice	Two-way RM ANOVA with Bonferroni post hoc test	Factor 1 (drug): F (1, 14) = 0.0025, P = 0.9608; Factor 2 (object): F (1, 14) = 38.59, P < 0.0001; Factor interaction: F (1, 14) =

	(17.69 ± 2.37%) in WT mice			0.6310, P = 0.4402.<br>Post hoc test:<br>p = 0.0037,<br>p = 0.0004
**Supplementary Fig. S6C, right panel**	Before CNO administration, Old (19.78 ± 2.60%) *vs.* New (34.82 ± 2.47%) in WT mice<br>After CNO administration, Old (14.75 ± 1.73%) *vs.* New (38.43 ± 2.79%) in WT mice	n $_{(WT)}$ = 8 mice	Two-way RM ANOVA with Bonferroni post hoc test	Factor 1 (drug): F (1, 14) = 0.0956, P = 0.7617; Factor 2 (object): F (1, 14) = 57.87, P < 0.0001; Factor interaction: F (1, 14) = 2.876, P = 0.1120.<br>Post hoc test:<br>p = 0.0019,<br>p < 0.0001
**Supplementary Fig. S6D, left panel**	Before CNO administration, M (34.34 ± 2.23%) *vs.* O (16.69 ± 2.01%) in *MECP2*-TG mice<br>After CNO administration, M (39.95 ± 2.89%) *vs.* O (13.73 ± 1.69%) in *MECP2*-TG mice	n $_{(MECP2\text{-TG})}$ = 8 mice	Two-way RM ANOVA with Bonferroni post hoc test	Factor 1 (drug): F (1, 14) = 0.5196, P = 0.4829; Factor 2 (object): F (1, 14) = 71.18, P < 0.0001; Factor interaction: F (1, 14) = 2.718, P = 0.1215.<br>Post hoc test:<br>p = 0.0006,<br>p < 0.0001
**Supplementary Fig. S6D, right panel**	Before CNO administration, Old (21.33 ± 2.77%) *vs.* New (23.45 ± 3.30%) in *MECP2*-TG mice<br>After CNO administration, Old (21.85 ± 3.12%) *vs.* New (27.03 ± 2.29%)	n $_{(MECP2\text{-TG})}$ = 8 mice	Two-way RM ANOVA with Bonferroni post hoc test	Factor 1 (drug): F (1, 14) = 0.4230, P = 0.5260; Factor 2 (object): F (1, 14) = 1.940, P = 0.1854; Factor interaction: F (1, 14) = 0.3408, P = 0.5687.<br>Post hoc test:

	in *MECP2*-TG mice			p > 0.9999, p = 0.3679.
**Supplementary Fig. S7B**	Cre (1.01 ± 0.01) *vs.* gRNA1 (1.07 ± 0.00); Cre (1.01 ± 0.01) *vs.* gRNA2 (0.99 ± 0.03); Cre (1.01 ± 0.01) *vs.* Cre + gRNA1 (0.45 ± 0.04); Cre (1.01 ± 0.01) *vs.* Cre + gRNA2 (0.17 ± 0.03).	$n_{Cre} = 3$; $n_{(gRNA1)} = 3$; $n_{(gRNA2)} = 3$; $n_{(Cre + gRNA1)} = 6$; $n_{(Cre + gRNA2)} = 6$	one way ANOVA with Bonferroni post hoc test	F (4, 16) = 129.2, p < 0.0001. Post hoc test: p > 0.9999; p > 0.9999; p < 0.0001; p < 0.0001
**Supplementary Fig. S7D**	Cre (1.01 ± 0.04) *vs.* gRNA1 (1.04 ± 0.03); Cre (1.01 ± 0.04) *vs.* gRNA2 (0.98 ± 0.05); Cre (1.01 ± 0.04) *vs.* Cre + gRNA1 (1.02 ± 0.04); Cre (1.01 ± 0.04) *vs.* Cre + gRNA2 (1.06 ± 0.05).	$n_{Cre} = 4$; $n_{(gRNA1)} = 4$; $n_{(gRNA2)} = 4$; $n_{(Cre + gRNA1)} = 8$; $n_{(Cre + gRNA2)} = 8$	one way ANOVA with Bonferroni post hoc test	F (4, 23) = 0.3558, p = 0.8373. Post hoc test: p > 0.9999, p > 0.9999, p > 0.9999, p > 0.9999.
**Supplementary Fig. S8B**	For scramble group, Empty 1 (E1) (20.85 ± 1.18) *vs.* Empty 2 (E2) (19.41 ± 2.97); For sgRNA1 group, E1 (19.95 ± 2.54) *vs.* E2 (18.44 ± 1.62); For sgRNA2 group, E1 (15.21 ± 1.19) *vs.* E2 (17.73 ± 1.98)	$n_{(Scramble)} = 8$ mice, $n_{(sg1)} = 10$ mice, $n_{(sg2)} = 10$ mice	Two-way RM ANOVA with Bonferroni post hoc test	Factor 1 (sgRNA): F (2, 25) = 1.949, P = 0.1634; Factor 2 (object): F (1, 25) = 0.0070, P = 0.9340; Factor interaction: F (2, 25) = 0.6391, P = 0.5362. Post hoc test: p > 0.9999, p > 0.9999,

				p > 0.9999
**Supplementary Fig. S8C**	For scramble group, object (O) (15.34 ± 2.69) *vs.* mouse (M) (28.27 ± 3.05);<br>For sgRNA1 group, O (15.90 ± 2.32) *vs.* M (29.64 ± 3.19);<br>For sgRNA2 group, O (14.89 ± 1.82) *vs.* M (27.36 ± 3.57)	$n_{(Scramble)}$ = 8 mice;<br>$n_{(sg1)}$ = 10 mice;<br>$n_{(sg2)}$ = 10 mice	Two-way RM ANOVA with Bonferroni post hoc test	Factor 1 (sgRNA): F (2, 25) = 0.2282, P = 0.7976;<br>Factor 2 (object): F (1, 25) = 25.88, P < 0.0001;<br>Factor interaction: F (2, 25) = 0.0222, P = 0.9780.<br>Post hoc test:<br>p = 0.0359,<br>p = 0.0107,<br>p = 0.0218
**Supplementary Fig. S9B**	WT (55.59 ± 4.63%) *vs.* *MECP2*-TG (47.55 ± 5.89%)	$n_{(WT)}$ = 11 mice;<br>$n_{(MECP2\text{-}TG)}$ = 13 mice	Unpaired Student's t test	t=1.04395, df=22,<br>p = 0.3078
**Supplementary Fig. S10A**	For WT group, MN (39.67 ± 6.20%) *vs.* ON (18.13 ± 3.46%);<br>NewN (27.92 ± 5.14%) *vs.* OldN (16.26 ± 3.39%);<br>For *MECP2*-TG group, MN (26.40 ± 4.27%) *vs.* ON (13.02 ± 3.49%);<br>NewN (22.66 ± 4.84%) *vs.* OldN (17.64 ± 3.15%),	$n_{(WT)}$ = 11 mice;<br>$n_{(MECP2\text{-}TG)}$ = 13 mice	Paired Student's t-test	t = 3.75163, df = 10, p = 0.0038;<br>t = 2.57629, df = 10,<br>p = 0.0276;<br>t = 3.38444, df = 12,<br>p = 0.0054;<br>t = 1.00158, df = 12,<br>p = 0.3363
**Supplementary Fig. S10B**	For CageN, WT (0.16 ± 0.02) *vs.* *MECP2*-TG (0.15 ± 0.01);	$n_{(WT)}$ = 11 mice;<br>$n_{(MECP2\text{-}TG)}$ = 13 mice	Outliers were identified by ROUT method and excluded in statistic	t = 0.46, df = 19,<br>p = 0.6542;<br>t = 1.14, df = 22,

	For MN, WT (0.23 ± 0.02) *vs.* *MECP2*-TG (0.20 ± 0.02); For ON, WT (0.17 ± 0.02) *vs.* *MECP2*-TG (0.18 ± 0.01); For NewN, WT (0.21 ± 0.02) *vs.* *MECP2*-TG (0.21 ± 0.03; For OldN, WT (0.20 ± 0.02) *vs.* *MECP2*-TG (0.19 ± 0.02)		analyze. Unpaired Student's t test	p = 0.2677; t = 0.37, df = 18, p = 0.7156; t = 0.16, df = 22, p = 0.8739; t = 0.95, df = 21, p = 0.3550
**Supplementary Fig. S13A**	For NewN excluding NewPN: WT New (0.81 ± 0.02) *vs.* WT Old (0.66 ± 0.02); *MECP2*-TG New (0.80 ± 0.02) *vs.* *MECP2*-TG Old (0.60 ± 0.02);	n (NewN - NewPN of WT) =219, n (NewN - NewPN of *MECP2*-TG) =138;	Two-way RM ANOVA with Bonferroni post hoc test	Factor 1 (genotype): F (1, 355) = 2.464, P = 0.1174; Factor 2 (object): F (1, 355) = 94.55, P < 0.0001; Factor interaction: F (1, 355) = 1.321, P = 0.2512. Post hoc test: p < 0.001; p < 0.001
	For OldN excluding OldPN: WT New (0.79 ± 0.02) *vs.* WT Old (0.75 ± 0.02); *MECP2*-TG New (0.72 ± 0.02) *vs.* *MECP2*-TG Old (0.66 ± 0.02); WT Old (0.75 ± 0.02) *vs.*	n (OldN - OldPN of WT) =127, n (OldN - OldPN of *MECP2*-TG) =118	Two-way RM ANOVA with Bonferroni post hoc test	Factor 1 (genotype): F (1, 243) = 12.02, P = 0.0006; Factor 2 (object): F (1, 243) = 5.208, P = 0.0234; Factor interaction: F (1, 243) = 0.3653, P = 0.5462. Post hoc test:

	MECP2-TG Old (0.66 ± 0.02)			p = 0.4559, p > 0.0923, p = 0.0063
**Supplementary Fig. S13B**	For NewN excluding NewPN: WT New (2.40 ± 0.05) *vs.* WT Old (2.25 ± 0.05); *MECP2*-TG New (2.35 ± 0.06) *vs.* *MECP2*-TG Old (2.39 ± 0.06);	n (NewN - NewPN of WT to new) =215, n (NewN - NewPN of WT to Old) = 200<br>n (NewN - NewPN of *MECP2*-TG to New) =138; n (NewN - NewPN of *MECP2*-TG to Old) =135;	Two-way ANOVA with Bonferroni post hoc test.	Factor 1 (genotype): F (1, 354) = 0.4627, P = 0.4968; Factor 2 (object): F (1, 330) = 0.9783, P = 0.3233; Factor interaction: F (1, 330) = 3.313, P = 0.0696.<br>Post hoc test: p = 0.0537, p > 0.9999
	For OldN excluding OldPN: WT New (2.23 ± 0.06) *vs.* WT Old (2.10 ± 0.07); *MECP2*-TG New (2.29 ± 0.07) *vs.* *MECP2*-TG Old (2.23 ± 0.07);	n (OldN - OldPN of WT to New) =123, n (OldN - OldPN of WT to Old) =122,<br>n (OldN - OldPN of *MECP2*-TG to New) =118, n (OldN - OldPN of *MECP2*-TG to Old) =115,	Two-way RM ANOVA with Bonferroni post hoc test	Factor 1 (genotype): F (1, 242) = 1.848, P = 0.1753; Factor 2 (object): F (1, 232) = 2.396, P = 0.123; Factor interaction: F (1, 232) = 0.3604, P = 0.5489.<br>Post hoc test: p = 0.2511, p > 0.9999
**Supplementary Fig. S13C**	For New social: WT NewPN (1.36 ± 0.07) *vs.* WT NewN excluding NewPN (1.45 ± 0.04); *MECP2*-TG NewPN (1.30 ± 0.10) *vs.* *MECP2*-TG	n (NewPN of WT to New) = 39, n (NewN - NewPN of WT to New) = 215; n (NewPN of *MECP2*-TG to New) = 28, n (NewN - NewPN of *MECP2*-TG to New) = 138;	Two-way RM ANOVA with Bonferroni post hoc test	Factor 1 (genotype): F (1, 416) = 0.0761, P = 0.7829; Factor 2 (object): F (1, 416) = 3.525, P = 0.0612; Factor interaction: F (1, 416) =

	NewN excluding NewPN (1.47 ± 0.05);			0.2657, P = 0.6065. Post hoc test: p > 0.9999, p = 0.7138,
	For Old social: WT OldPN (1.47 ± 0.15) *vs.* WT OldN excluding OldPN (1.33 ± 0.04); *MECP2*-TG OldPN (1.29 ± 0.09) *vs.* *MECP2*-TG OldN excluding OldPN (1.50 ± 0.05)	n $_{\text{(OldPN of WT to Old)}}$ = 19; n $_{\text{(OldN - OldPN of WT to Old)}}$ = 122; n $_{\text{(OldPN of } MECP2\text{-TG to Old)}}$ = 26; n $_{\text{(OldN - OldPN of } MECP2\text{-TG to Old)}}$ = 115	Two-way RM ANOVA with Bonferroni post hoc test	Factor 1 (genotype): F (1, 278) = 0.0053, P = 0.9423; Factor 2 (object): F (1, 278) = 0.1198, P = 0.7295; Factor interaction: F (1, 278) = 4.725, P = 0.0306. Post hoc test: p > 0.9999, p = 0.3456
**Supplementary Fig. S14A**	NewPN (1.60 ± 0.65) *vs.* OldPN (3.70 ± 1.38)	n = 10 mice	Paired Student's t-test	t = 1.378, df = 9, p = 0.2014
**Supplementary Fig. S14C**	For P_{up}: New (0.65 ± 0.04) vs Old (0.42 ± 0.07);	n = 16 neurons	Paired Student's t-test	t = 3.581, df = 15, p = 0.0027
	For $Amplitude_{up}$: New (1.13 ± 0.12) vs Old (0.87 ± 0.07);	n = 11 neurons	Paired Student's t-test	t = 3.019, df = 10, p = 0.0129
	For Time to $peak_{up}$: New (2.04 ± 0.15) vs Old (1.43 ± 0.24)	n = 12 neurons	Paired Student's t-test	t = 2.219, df = 11, p = 0.0485
**Supplementary Fig. S14D**	For P_{up}: New (0.44 ± 0.03) vs Old (0.56 ± 0.04)	n = 34 neurons	Paired Student's t-test	t = 2.885, df = 33, p = 0.0068

	For Amplitude$_{up}$: New (0.97 ± 0.07) vs Old (1.03 ± 0.09)	n = 34 neurons	Wilcoxon matched-pairs signed rank test	W = 125, p = 0.2932
	For Time to peak$_{up}$: New (1.79 ± 0.13) vs Old (1.45 ± 0.06)	n = 33 neurons	Paired Student's t-test	t = 1.522, df = 32, p = 0.1377
**Supplementary Fig. S14E**	For New Social Epochs: WT (2.16 ± 0.10) vs *MECP2*-TG (2.53 ± 0.14) vs *MECP2*-TG -res (2.16 ± 0.13)	n (WT) = 39 neurons, n (*MECP2*-TG) = 28 neurons, n (*MECP2*-TG-res) = 15 neurons	Ordinary one-way ANOVA with Bonferroni's post hoc test	F (2, 79) = 0.7416, P = 0.0470; post hoc test: p (WT vs *MECP2*-TG) = 0.0593, p (WT vs *MECP2*-TG-res) > 0.9999, p (*MECP2*-TG vs *MECP2*-TG-res) = 0.2191
	For Old Social Epochs: WT (1.76 ± 0.13) vs *MECP2*-TG (2.57 ± 0.14) vs *MECP2*-TG -res (1.76 ± 0.26)	n (WT) = 34 neurons, n (*MECP2*-TG) = 27 neurons, n (*MECP2*-TG-res) = 15 neurons	Ordinary one-way ANOVA with Bonferroni's post hoc test	F (2, 73) = 8.5960, P = 0.0004; post hoc test: p (WT vs *MECP2*-TG) = 0.0008, p (WT vs *MECP2*-TG-res) > 0.9999, p (*MECP2*-TG vs *MECP2*-TG-res) = 0.0082
**Supplementary Fig. S14F**	For New Social Epochs: WT (1.99 ± 0.23) vs *MECP2*-TG (2.08 ± 0.10) vs *MECP2*-TG -res (1.79 ± 0.13)	n (WT) = 15 neurons, n (*MECP2*-TG) = 28 neurons, n (*MECP2*-TG-res) = 33 neurons	Ordinary one-way ANOVA with Bonferroni's post hoc test	F (2, 73) = 1.386, P = 0.2567; post hoc test: p (WT vs *MECP2*-TG) > 0.9999, p (WT vs *MECP2*-TG-res) > 0.9999, p (*MECP2*-TG vs *MECP2*-TG-res) = 0.3220

	For Old Social Epochs:<br>WT (1.35 ± 0.21) vs *MECP2*-TG (2.40 ± 0.12) vs *MECP2*-TG -res (1.45 ± 0.16)	n (WT) = 19 neurons, n (*MECP2*-TG) = 26 neurons, n (*MECP2*-TG-res) = 33 neurons	Ordinary one-way ANOVA with Bonferroni's post hoc test	F (2, 75) = 12.52, P < 0.0001;<br>post hoc test:<br>p (WT vs *MECP2*-TG) = 0.0002, p (WT vs *MECP2*-TG-res) > 0.9999, p (*MECP2*-TG vs *MECP2*-TG-res) = 0.0001

Suppl. Table S2: The information of antibodies and viruses

REAGENT or RESOURCE	SOURCE	IDENTIFIER
**Antibody**		
Rabbit anti-MECP2	Cell Signaling Technology	Cat#3456S; RRID: AB_2143849
Mouse anti-Cre Recombinase	Millipore	Cat# MAB3120; RRID: AB_2085748
Mouse anti-β-actin	Sungene Biotech	Cat# KM9001T
Goat anti- Rabbit Alexa Fluor 488	Biotium	Cat#20012; RRID: AB_10559670
Goat anti- mouse Alexa Fluor 488	Biotium	Cat#20010; RRID: AB_10559812
Donkey anti-Rabbit Alexa Fluor 633	Biotium	Cat#20125; RRID: AB_10557270
**Virus**		
rAAV2/9-hSyn-GCaMP6s-WPRE-pA	BrainVTA Co., China	Cat#PT0145
rAAV2/9-EF1a-DIO-GCaMP6s-WPRE-pA	BrainVTA Co., China	Cat#PT0071
rAAV-cfos-tTA-WPRE-pA	BrainVTA Co., China	Cat#PT3022
rAAV-TRE3G-DIO-hM4d(Gi)-mcherry-WPRE-pA	BrainVTA Co., China	Cat#PT0036
rAAV-TRE3G-DIO-mcherry-WPRE-pA	BrainVTA Co., China	Cat#PT0113
LentiV-Cas9-puro	Gift from Xiaotao Duan Lab	N/A
pCAG-mCherry-Cre	Gift from Sbo-bio	N/A
AAV2-hSyn-mCherry-WPRE-pA	Obio Technology Co, Shanghai	Cat#H3193
AAV2-U6-loxP-MECP2-gRNA-EF1a-mCherry	BrainVTA Co., China (Gift from Yichang Jia Lab)	N/A

Suppl. Table S3: The information of software and algorithms

REAGENT or RESOURCE	SOURCE	IDENTIFIER
**Software and Algorithms**		
MATLAB	Mathworks	https://www.mathworks.com/products.html, RRID: SCR_001622
ImageJ	NIH	https://imagej.nih.gov/ij/index.html, RRID: SCR_003070
GraphPad Prism	GraphPad Software	https://www.graphpad.com/scientific-software/prism/, RRID: SCR_002798
python	Python.org	https://www.python.org/, RRID:SCR_008394
yolo-v3	Reference (*55*)	N/A
Fiji	NIH	https://fiji.sc/ Reference (*59*)
DeepLabCut	Reference (*56*)	N/A
FHIRM-TPM V2.0	Transcend Vivoscope Biotech Co., Ltd, China	http://tv-scope.com/
GINKGO-MTPM	Transcend Vivoscope Biotech Co., Ltd, China	http://tv-scope.com/
Custom-developed neuron identification framework	This work	N/A
Custom MATLAB scripts for calcium signal analysis	This work	N/A
Modified Venn diagram	Flanders Interuniversity Institute for Biotechnology	http://bioinformatics.psb.ugent.be/webtools/Venn/
CRISPick design tool	Broad Institute	https://portals.broadinstitute.org/gppx/crispick/public

Movie S1. An example video demonstrating PrL neuron activity during social investigation of mouse and object. Raw grayscale video (motion corrected, Top) and the behavioral video (Bottom) on the left and the calcium activity trace of related cell on the right.

Movie S2. An example video demonstrating PrL pyramidal neuron activity during social investigation of mouse and object. Raw grayscale video (motion corrected, Top) and the behavioral video (Bottom) on the left and the calcium activity trace of related cell on the right.

Movie S3. An example video demonstrating PrL GABAergic neuron activity during social investigation of mouse and object. Raw grayscale video (motion corrected, Top) and the behavioral video (Bottom) on the left and the calcium activity trace of related cell on the right.

Movie S4. An example video demonstrating activity of NewPN of WT mice during social novelty preference stage. Raw grayscale video (motion corrected and cropped, Left) and the behavioral video (Right) on the top and the calcium activity trace of related cell on the bottom.

Movie S5. An example video demonstrating activity of OldPN of WT mice during social novelty preference stage. Raw grayscale video (motion corrected and cropped, Left) and the behavioral video (Right) on the top and the calcium activity trace of related cell on the bottom.

Movie S6. An example video demonstrating activity of NewPN of *MECP2*-TG mice during social novelty preference stage. Raw grayscale video (motion corrected and cropped, Left) and the behavioral video (Right) on the top and the calcium activity trace of related cell on the bottom.

Movie S7. An example video demonstrating activity of OldPN of *MECP2*-TG mice during social novelty preference stage. Raw grayscale video (motion corrected and cropped, Left) and the behavioral video (Right) on the top and the calcium activity trace of related cell on the bottom.
